# Canopy insect communities are shaped by the genes and phenotypes of their aspen hosts

**DOI:** 10.1371/journal.pone.0327554

**Published:** 2025-07-17

**Authors:** Clay J. Morrow, Jennifer Lind-Riehl, Christopher T. Cole, Kennedy Rubert-Nason, Cécile Ané, Richard L. Lindroth

**Affiliations:** 1 Department of Forest and Wildlife Ecology, University of Wisconsin-Madison. Madison, Wisconsin, United States of America; 2 Department of Entomology, University of Wisconsin-Madison. Madison, Wisconsin, United States of America; 3 Department of Statistics, University of Wisconsin-Madison. Madison, Wisconsin, United States of America; Helmholtz Centre for Environmental Research - UFZ, GERMANY

## Abstract

1. Community genetics research aims to identify genetic and phenotypic mechanisms that shape communities as extended phenotypes. To date, most progress has been made identifying variation in herbivore communities associated with intraspecific variation in plants, with little focus on identifying specific genes or traits responsible for that variation. Here, we identify how extended phenotype variation of a foundation tree species, *Populus tremuloides*, arises from trait variation among individuals and specific genes. 2. We quantified heritability for 13 tree traits -- including phenology, defense chemistry, reproduction, and morphology -- and for 18 associated insect species (640,557 individuals). We performed genomic association analyses to identify genetic links to heritable traits and insects. 3. We found that both tree traits and communities of insect herbivores were highly heritable, and that structure and diversity of insect communities responded to heritable aspen traits. The most heritable insects were leaf-modifying specialist herbivores. We identified 73 genes associated with tree traits linked to insect communities and an additional 15 genes associated directly with insect community composition. 4. By linking intraspecific variation to community composition and structure through probable genomic mechanisms, this work demonstrates the salience of the genes-to-ecosystems paradigm in plant-insect systems.

## Introduction

Intraspecific trait variation can have far-reaching consequences that extend beyond populations to communities and ecosystems. On average, 25% of the total trait variation within plant communities is attributable to intraspecific variation [1]. The ecological impacts of intraspecific variation can be comparable to those of interspecific variation [2,3], especially in plants [4]. For example, intraspecific variation in expression of secondary metabolites influences herbivore abundance and performance (e.g., [5,6]) that can ultimately lead to differences in diversity and structure of associated insect communities [7–9]. Many other studies have similarly shown links between genetic variation and communities of arthropods and fungi (e.g., [10–15]), which in turn have consequences for ecosystem function and eco-evolutionary dynamics. The differential effects of plant genotypes on their environment are known as extended phenotypes [16,17]. Over the last decade, a growing number of studies have explored genetic variation underlying extended phenotypes, especially in plant systems [18] where the effects appear common and ecologically relevant.

Although research addressing intraspecific variation and its genetic underpinnings is growing [18], few studies have investigated those connections directly [19], especially for extended phenotypes. A major criticism of this research field is that experimental genotypes are often selected to maximize phenotypic variation, but may not be representative of natural diversity [19–21]. Similarly, minimal research has investigated which genes govern ecologically relevant intraspecific variation and extended phenotypes. Understanding not only how associated communities differ among plant genotypes, but also the roles that genes and traits play, can highlight the importance of genomic variation in maintaining diverse and resilient ecosystems. Very few studies have demonstrated a connection between genes, traits, and ecosystems, especially in the context of community dynamics [21,22]. Discoveries of genetic links to communities have important conservation implications, since phenotypic and genetic variation support ecosystem health [23,24]. Better understanding of these mechanisms could, for example, allow forest managers to select for populations with genetic composition that promotes community stability and resilience, or resistance to herbivore defoliators.

Ecologically-relevant extended phenotypes are most likely to occur within species that have high levels of heritable trait variation, are associated with diverse communities, and have relatively large phenotypically mediated impacts on those communities [19,25]. Trembling aspen (*Populus tremuloides* Michx.) and related *Populus* species meet those criteria: they exhibit substantial variation across a wide range of traits, many of which are genetically heritable [26]; they are associated with biologically diverse communities that are also heritable [25,27]; and much of their considerable phenotypic diversity occurs in ecologically relevant traits that shape those associations [28,29]. Indeed, these characteristics in *P. tremuloides* explain its role as a foundation species [30] throughout its expansive North American range [31,32]. Aspen and other *Populus* have also been important models in plant-insect interaction research, in part due to their quantitative variation in chemical defense [29,33]. Because *Populus* is a model organism for plant genomic research [34,35], a wealth of quality genomic information is available.

This research aimed to elucidate relationships between intraspecific plant variation and insect communities. Our work used an experimental population (common garden) of replicated aspen genets, extending previous studies of that system [36,37] with improved genotyping methods, more biological replicates, and improved genomic association models. Our group’s previous work in this domain documented one of the first clear cases of genomic variation linked to community phenotypes [22]. The work we report here aimed to refine and improve exploration into these relationships. First, in contrast to the immature trees studied previously, this work was conducted with a population more representative of a natural aspen stand: the trees had reached sexual and chemical maturity, and the canopy had fully closed. Second, we employed more advanced methodological, genomic, and statistical techniques to increase our ability to detect genetic patterns.

We first explored whether insect communities are an extended aspen phenotype by asking “are communities of aspen-associated insects genetically heritable?” We expected insect abundance, diversity, and community composition to vary among aspen genets, especially for specialist insects. We then investigated the role that tree phenotypes play in those relationships by asking “do heritable aspen traits contribute to shaping insect communities?” We expected that the tree traits we measured would be associated with insect communities, but that effects would differ among insect species. Finally, we assessed the potential genomic drivers by asking “do specific aspen genes contribute to shaping insect communities?” We expected that some genes would be identified as potential drivers of insect community structure but had no *a priori* expectations about specific genes because the relationship of aspen traits to specific gene function is complex and largely unknown.

## Materials and methods

### Experimental aspen population

The aspen population consisted of 1,568 experimental trees representing 492 genets. The common garden was established with sprouts propagated from roots collected from genets occurring along a north-south gradient in Wisconsin U.S.A. (358 km latitude range, 186 km longitude range). In 2010, trees were planted with 2.5 × 2.5 m spacing in an incomplete randomized block design with four blocks. Trees from the outer perimeter of the garden were excluded from the study to guard against edge effects and are not considered experimental trees. The garden is located at the University of Wisconsin-Madison Arlington Agricultural Research Station (43.32°N, 89.33°W). More detailed information about the garden is provided by Barker et al. [36,38]. This experimental population was established with the express purpose of simulating naturally occurring genetic variation while reducing the influence of environmental factors. As such, the garden exhibits substantial phenotypic variation in diverse traits, including growth, chemical defense, reproduction, and phenology [26], and has been colonized by a diverse assemblage of tree-feeding insects [36].

### Herbivore communities: extended phenotype

Over the course of two years, we conducted four total insect surveys to quantify herbivore communities occurring on individual aspen trees, to compare the communities among genets, and to correlate them with tree traits. Due to the large number and size of trees in the garden and a desire to minimize temporal variation within surveys, we restricted sampling to the lower third of the canopy. We counted and identified insect herbivores to the smallest possible taxonomic unit. We also surveyed ants because of their expected relationships with aphids and extrafloral nectaries. Trees were surveyed for durations proportional to their diameter, up to a maximum of 10 minutes, such that approximately 75% of the sampling area was surveyed for each tree. These thresholds were informed by preliminary evaluation of a random subset of trees one week prior to each survey. Surveys occurred in mid-June and early-August of 2016 and 2017. We surveyed north to south, by experimental block, and each survey lasted ten days.

We quantified diversity and composition of insect communities for each tree in the garden. We calculated species abundance, species richness, species evenness, Shannon index, and community ordination axes (non-metric multidimensional scaling, NMDS) as metrics of diversity [39]. Species incidence (presence/absence) served as our composition metrics. NMDS ordination was performed on community abundance using Bray-Curtis dissimilarities [40,41], four dimensions, and with resulting stress of 0.135. This research focused on only common insects (i.e., those observed on at least five percent of trees) to reduce the likelihood of spurious correlations. Insects were also categorized into functional groups, only two of which were common and included in analyses: free-feeding insects and leaf-modifying insects. The latter group contained leaf-miners, leaf-gallers, and leaf-rollers.

### Aspen traits: phenotype

We quantified aspen traits that, based on previous research (e.g., [36,38]), were expected to influence insect communities: phytochemistry, leaf morphology, tree size, tree phenology, and reproduction. Immediately following each insect survey, we collected leaves for measurements of phytochemical and morphological traits. We collected four leaves, semi-randomly, from each cardinal direction of the lower canopy (16 leaves per tree). Leaves were digitally scanned, vacuum dried, weighed, ground with a ball mill, and aliquoted for chemical analyses. We included four phytochemical metrics in this study: condensed tannin (CT), salicinoid phenolic glycoside (SPG), and nitrogen (N) concentrations, and carbon-nitrogen ratio (C:N). We extracted CTs into acetone and quantified them via the acid butanol assay [42], using standards purified from aspen following Hagerman and Butler [43]. We extracted SPGs into methanol and quantified them via UPLC-mass spectrometry [44]. Commercially available salicin and lab-purified salicortin, tremulacin, and tremuloidin (Sigma-Aldrich) served as standards. We report total SPGs as the sum of those four compounds. Salicortin and tremulacin comprised >90% of the total SPG pool; details on quantitative variation in individual compounds are provided by Cole et al. [26]. A ThermoFlash carbon/nitrogen elemental analyzer was used to determine N and C:N. All chemical concentrations are expressed as a percentage of dry leaf weight (% dw). Using a LI-COR 3100 area meter, we calculated average leaf area (ALA; total leaf area divided by number of leaves) and specific leaf area (SLA; total leaf area divided by total leaf mass). Using digital leaf scans, we quantified the density of extrafloral nectaries (EFN; total number of nectaries divided by number of leaves).

Unlike leaf traits, tree size, phenology, and reproduction were quantified only once per year. We measured tree diameter (D 1.4 m above ground level) in centimeters and tree height (h) in meters at the end of each growing season. From these measurements, we calculated basal area (BA; $\pi {\left(\frac{D}{2}\right)}^{2}$) and approximated tree volume ($D^{2}h$). At the beginning of each growing season, we tracked bud set stage and used polynomial regression to estimate onset of budbreak in degree days [37,45]. We also counted the number of twigs possessing flowers as a measure of reproduction. Because aspen trees flower early in the growing season, utilizing resources stored during the previous season (i.e., a lag effect), we counted flowers in the year following each insect survey.

### Aspen genomics: Genotype

To associate phenotypic traits with genetic variation in our population, we conducted a genome association analysis. We quantified genomic variation for each genet in the common garden using sequence capture genotyping [46]. Sequence data were aligned to the *Populus tremula* v1.1 [37] genome assembly. Following sequencing, single nucleotide polymorphisms (SNPs) were identified and variant filtering was conducted to ensure the quality of the genomic data. Specifically, we removed all SNPs with more than 30% missing data. Genotypes with more than 20% missing data were also removed. A total of 113,674 SNPs remained after applying a minor allele frequency cut-off of 5% for genomic association analyses. All genomic sequencing, alignment, variant calling, and filtering procedures are detailed by Riehl et al. [37] and Barker et al. [36].

We also used genomic information to verify genet identity and sex. Genetic markers obtained via unique sequence repeat microsatellite genotyping (SSR) [47,48] were evaluated with GenAlEx [49,50] and verified with SNP and phenotype data. Subsequently, genotype identity of a small number of trees (n=67) was updated relative to previous studies conducted in this garden [36,38]. Sex was determined from a *Populus* sex marker TOZ19 [51] and verified, when possible, with flower morphology.

### Statistical analysis

To ascertain the degree to which aspen phenotypes and extended phenotypes were mediated by genetics, we estimated the relative contributions of genetic variation to trait expression and insect community variation within the aspen population. We measured heritability of aspen phenotypes and community metrics by partitioning variance components of random effects regression models. We parameterized these models with only random effects as independent variables. Along with genet identity, we also included experimental block, survey year, survey month, and distance from the plot border (edge distance) as environmental factors. This parameterization allowed us to separate genetic variation from environmental variation. Due to the strong influence of ontogeny on tree traits [26], we also included tree age as a covariate in phenotype models. We then calculated broad-sense heritability ($H^{2}$) by dividing the genotype-associated variance component by the total phenotypic variance of the population (method adapted from Barker et al. [36]). Linear random effects models were used to determine heritability of most traits, but one set of community metrics (species incidence) was fit with logistic mixed effects models because of the binomial nature of this variable. In all cases, we bootstrapped estimates of $H^{2}$ 1000 times to obtain the mean estimate (${\hat{H}}^{2}$) and 95% confidence bounds. For the purposes of this study, we consider a phenotype or extended phenotype highly heritable, *relative to other traits*, when at least 50% of the variation in that characteristic is explained by genotype (i.e., ${\hat{H}}^{2}\geq 0.5$). We consider a characteristic moderately heritable when between 20 and 50% of the variation is explained by genotype ($0.2\leq {\hat{H}}^{2}<0.5$) and weakly heritable if less than 20% is explained $\left({\hat{H}}^{2}<0.2\right)$. Characteristics with lower 95% confidence bounds ≤0 are considered non-heritable.

We used mixed effects models and redundancy analysis to determine the influence of aspen phenotype in shaping heritable components of insect communities. We performed exhaustive model selection to determine which traits to include (based on lowest AIC) as independent variables (fixed effects) in models of insect metrics. Experimental block, survey year, survey month, genet identity, and edge distance were included as random effects in all models to account for environmental variation. In all analyses pertaining to BA, a square root transformation was used (BAsqrt) to meet model assumptions. Volume and C:N were highly correlated with BAsqrt (r=0.94) and N (r=0.95), respectively. Thus, these redundant variables were omitted from statistical models to avoid multicollinearity. We report the standardized effect sizes of tree traits on community metrics, as derived from the chosen models. We also used redundancy analysis (RDA), a multivariate regression tool, and permutational analysis of variance (PERMANOVA) to evaluate the effects of aspen traits on herbivore community structure.

We then conducted genomic association analyses of tree traits and insect communities to identify underlying genes with links to community characteristics, using the full SNP set. We fit mixed effects models for each combination of SNPs and response variables (i.e., phenotype and extended phenotype). SNP genotype comprised the sole fixed effect in these models. Experimental block, survey year, survey month, genet identity, and distance to edge again comprised the random effects. Models incorporating insect responses were fit with and without the explanatory tree traits as covariates. This approach allowed us to determine the degree to which the phenotypes mediate the relationships between genes and extended phenotypes. We compared these models with those used in our previous work [36,37] by utilizing a simulated aspen garden with similar genetic and phenotypic conditions, extended phenotypes, and known relationships (see method summary in S1 File). The selected mixed effects model outperforms the traditional method, which has a substantially inflated Type I error rate and identifies associations that do not exist (S1 File). Thus, the new model is more conservative, lending high confidence to any detected genomic links to extended phenotypes. A Wald chi-squared test was used to determine the significance of SNP effects on responses and we performed multiple-test corrections by calculating Storey’s false discovery rate, q [52]. We set a significance threshold of q<0.15 for identifying SNP associations. At this level, it is expected that fewer than 15% of the associations detected are false positives. Once significant SNPs were identified, we investigated known functions of the genes in which these variants were located. Additionally, gene set enrichment analyses [53] were conducted for the top 0.1% of SNPs (determined by uncorrected p-value). Gene enrichment can identify whether clusters of functionally related genes are associated with traits, even if each individual gene association was too weak to detect.

We also evaluated the probability that genomic associations may exist with community components, independent of whether they were detected by our models. This probability was evaluated via Tukey’s higher criticism test of p-value distributions [54,55]. The higher criticism statistic (HC) was calculated as the square root of the number of tests (*n*) multiplied by the difference between the proportion of significant tests observed (ϕ) and the proportion expected if no associations exist, divided by the standard error (eq. 1). We then evaluated significance, for each response, by comparing the HC statistic to the standard normal distribution [55].


$$HC=\sqrt{n}\frac{\phi -0.05}{\sqrt{0.05\times 0.95}}$$ (1)

All statistical analyses were conducted using the *R* statistical software library [56]. Mixed models were fit using tools from the *lme4* package [57] and RDA, NMDS, and PERMANOVA were conducted using the *vegan* package [40].

## Results

### Heritability of aspen-associated insect communities

**Fig 1:**
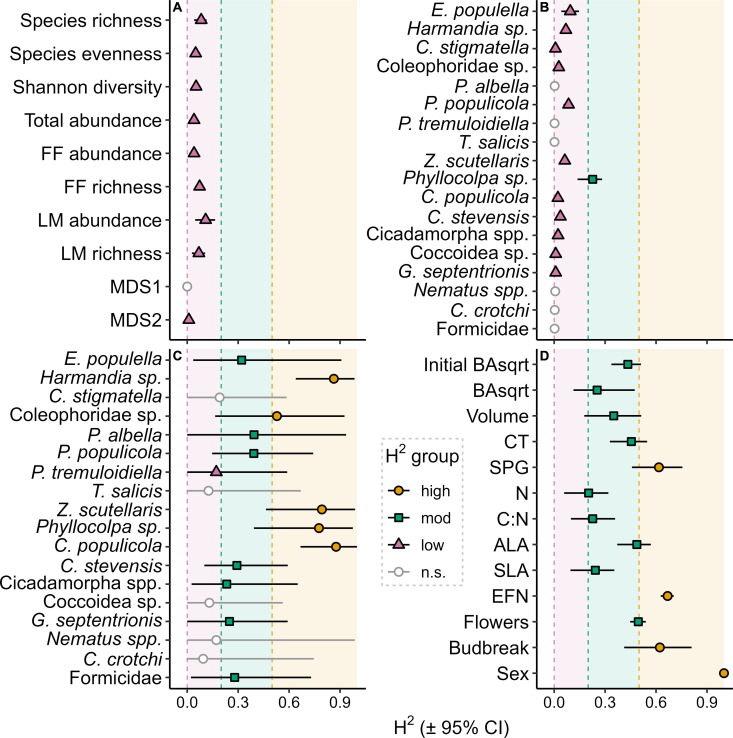
Bootstrapped broad-sense heritability (H^2^) estimates and asymmetrical 95% confidence intervals. Phenotype and extended phenotype along the y-axis are grouped into (A) community diversity metrics, (B) individual species abundance, (C) individual species incidence, and (D) aspen traits. Colors and point shapes indicate the heritability levels: high, moderate (mod), low, and non-heritable (n.s.). Abbreviations are FF: free feeding insects, LM: leaf modifying insects, MDS: community multi-dimensional scaling axis, BAsqrt: square root of basal area, CT: condensed tannins, SPG: salicinoid phenolic glycosides, N: nitrogen, C:N: carbon-nitrogen ratio, ALA: average leaf area, SLA: specific leaf area, and EFN: extrafloral nectaries. Initial BAsqrt represents tree size in 2012.

Aspen-associated insect communities were diverse. We observed over 100 insect species and upwards of 650,000 individual insects during this study. Of these, 18 species occurred commonly in each of the four insect surveys (Table 1), for a total of 640,557 individual insects. Of the common insects, at least 14 were specialists of *Populus* or Salicaceae. Seven species were highly mobile, free-feeding herbivores and ten were largely sessile, leaf-modifying herbivores (i.e., gallers, miners, and rollers). The remaining common insects were aphid-tending ants. In general, correlations among abundances of different species were relatively low (Fig 1S in S2 File) but exhibited some interesting and expected patterns (e.g., between ants and aphids, and within functional groups).

**Table 1:** Common insects identified during surveys. Column descriptions: Insect species, family, functional group, diet breadth, Tukey’s higher criticism statistic, and associated P-value. Note that Cicadamorpha spp. is a polyphyletic group containing functionally similar species from Cicadellidae and Membracidae and that the vast majority of Nematus spp. were one morphospecies, likely *Nematus oligospilus* [58].

Insect	Family	Functional group	Diet breadth	HC	P
*Ectoedemia populella*	Nepticulidae	Leaf modifier	*Populus* [59]	4.302	<0.001
*Harmandia sp.*	Olacaceae	Leaf modifier	*Populus* [60]	4.853	<0.001
*Caloptilia stigmatella*	Gracillariidae	Leaf modifier	Salicaceae [61]	−1.224	0.890
Coleophoridae *sp.*	Coleophoridae	Leaf modifier	unknown	2.608	0.005
*Paraleucoptera albella*	Lyonetiidae	Leaf modifier	*Populus* [62]	0.648	0.259
*Paraphytomyza populicola*	Agromyzidae	Leaf modifier	*Populus* [63]	0.846	0.199
*Phyllonorycter tremuloidiella*	Gracillariidae	Leaf modifier	*Populus*, *Salix* [64]	1.167	0.122
*Tachyerges salicis*	Curculionidae	Leaf modifier	Salicaceae [65]	−0.394	0.653
*Zeugophora scutellaris*	Megalopodidae	Leaf modifier	*Populus*, *Salix* [66]	4.809	<0.001
*Phyllocolpa sp.*	Tenthredinidae	Leaf modifier	*Populus*, *Salix* [67]	2.344	0.010
*Chaitophorus populicola*	Aphididae	Free feeder	*Populus* [68]	5.328	<0.001
*Chaitophorus stevensis*	Aphididae	Free feeder	*Populus* [68]	1.505	0.067
Cicadamorpha spp.	N/A	Free feeder	unknown	1.137	0.128
Coccoidea *sp.*	Coccoidea	Free feeder	unknown	1.514	0.065
*Gluphisia septentrionis*	Notodontidae	Free feeder	*Populus* [69]	1.665	0.048
*Nematus spp.*	Tenthredinidae	Free feeder	Species specific [70], likely Salicaceae [58]	2.246	0.012
*Chrysomela crotchi*	Chrysomelidae	Free feeder	Salicaceae [71]	1.614	0.053
Formicidae	Formicidae	Aphid tender		0.028	0.489

Aspen exhibited substantial intraspecific variation in their associated insect communities and the composition of those communities was largely heritable, whereas broader diversity metrics and abundance were less so. Species occurrence was highly variable within the population (Fig B in S2 File); on average, variation among aspen genotypes accounted for 28% of the total variation in insect community composition, as assessed with species incidences. Heritability was high for the incidence of five specialist insects: *Harmandia sp.*, Coleophoridae *sp.*, *Zeugophora scutellaris*, *Phyllocolpa sp.*, and *Chaitophorus populicola* (${\hat{H}}^{2}\;$0.53–0.88; Fig 1C). Seven additional species, *Ectoedemia populella*, *Paraleucoptera albella*, *Paraphytomyza populicola*, *Chaitophorus stevensis*, Cicadamorpha *spp., Gluphisia septentrionis*, and Formicidae *spp.* were moderately heritable (${\hat{H}}^{2}$ 0.23–0.39; Fig 1C). One species, *Phyllonorycter tremuloidiella,* was weakly heritable (${\hat{H}}^{2}=0.17$) and the remaining five common species were non-heritable (Fig 1C).

Diversity of insect communities was also variable across trees and genets within the population (e.g., richness ranged from 0–14 species with an average of 5 species; Fig C in S2 File). Species abundances were similarly variable (Fig D in S2 File). All diversity metrics were weakly heritable (${\hat{H}}^{2}<0.2$, Fig 1A) except the primary multivariate community axis (MDS1), which was non-heritable. On average, genotype accounted for only 7% of the variation in diversity metrics. Intraspecific effects on abundance of individual insect species were also weak overall: eleven common species were weakly heritable while six were non-heritable (Fig 1B). Uniquely, *Phyllocolpa sp.* abundance was moderately heritable (${\hat{H}}^{2}=0.23$, Fig 1B). Genotype accounted for only a small amount of the variation in common insect abundance overall (6%). Due to the uniformly low heritability of diversity and abundance metrics, they were not of interest as extended phenotypes and were excluded from further analyses except where otherwise indicated.

### Tree traits and effects on insect communities

**Fig 2:**
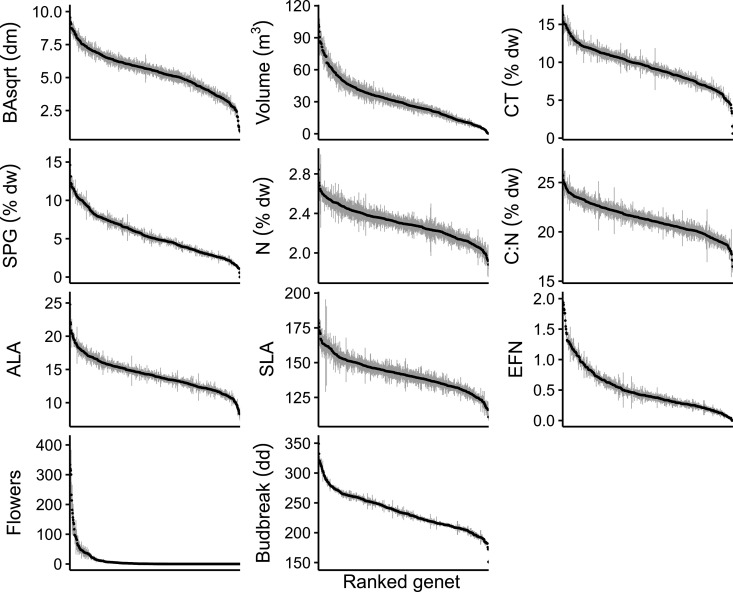
Variation in ecologically relevant aspen traits among aspen genets. Points represent the average trait values for a genet and grey error bars represent ± 1 standard deviation. Panels correspond to the different aspen traits.

Aspen phenotypic traits were highly variable, and most were strongly heritable (Fig 1D). Tree size, phytochemical composition, leaf morphology, reproduction, and phenology all varied substantially among genotypes within the population (Fig 2). Condensed tannin concentration was the most variable trait (52-fold range), whereas nitrogen concentration was the least variable (2.4-fold range) (Fig 2). All tree traits exhibited significant genotypic variation and were at least moderately heritable (${\hat{H}}^{2}>0.2$, Fig 1D). Four traits – salicinoid phenolic glycoside concentration, extrafloral nectary density, budbreak timing, and flower production – were highly heritable (${\hat{H}}^{2}$ 0.50–0.67). Both ALA and CT concentration nearly met the highly heritable classification (${\hat{H}}^{2}$ of 0.49 and 0.46, respectively). We excluded sex from our heritability classifications because it is completely genetically mediated (i.e., ${\hat{H}}^{2}=1$, Fig 1D) and, therefore, invariant within genets.

**Fig 3:**
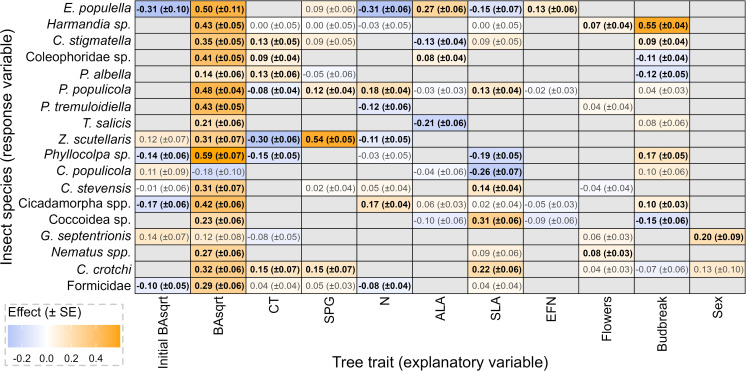
Standardized effects ( ± standard error) of aspen traits on insect incidence, derived from the best mixed-effects logistic regression models (selected by AIC) for each species. Blank cells indicate that a trait was not included in the selected model. Black bolded values indicate statistically significant effects (P < 0.05) while grey effects are non-significant. All models also included tree age and location within the garden as fixed covariates as well as genet identity and time as random covariates. Abbreviations are BAsqrt: square root of basal area, CT: condensed tannins, SPG: salicinoid phenolic glycosides, N: nitrogen, ALA: average leaf area, SLA: specific leaf area, and EFN: extrafloral nectaries. Initial BAsqrt represents trees size in 2012.

These phenotypic traits were also strongly associated with the composition of insect communities, as represented by species incidence (see Fig E in S2 File). In the best models of species incidence (Fig 3), BAsqrt was positively associated with all but two insect species. The exceptions were *C. populicola and G. septentrionis*, which were not significantly associated with tree size. Effects of CT concentrations were mixed: incidence of three species was decreased by CTs while incidence of four species was increased. Somewhat surprisingly, increased SPG concentrations resulted in *increased* incidence of three species and decreased incidence of none. Foliar N concentrations, ALA, and SLA each exhibited positive and negative effects on many common insects. CTs, SPGs, and SLA were all included as explanatory factors in the best model of *Harmandia sp.* incidence, but the magnitudes of these effects were too small to be functionally relevant (effect size less than 0.001). EFN density increased incidence of one species. Flower production was weakly associated with occurrence of two species. Budbreak timing influenced seven species and was negatively associated with three of them. Notably, budbreak timing that was delayed by one standard deviation (32.5 degree days) resulted in a 55% increased probability of *Harmandia* sp. occurrence. Male aspen had increased incidence of one species, *G. septentrionis*, relative to females.

Aspen phenotype, especially size and condensed tannins, was associated with insect community diversity and abundance metrics. Tree size (BAsqrt) was most strongly correlated with species richness (Pearson’s r=0.29) and, among functional groups, was most strongly correlated with richness of free-feeding insects (r=0.25). Conversely, condensed tannin concentration was negatively correlated with species richness (r= −0.16), primarily affecting leaf-modifying insects (r= −0.16). CTs were also negatively associated with abundance of leaf-modifying insects (r= −0.22) and were positively correlated with species evenness (r=0.16). SLA was not strongly associated with any diversity metrics, but ALA was associated with abundance of leaf-modifying insects (r=0.16). All other measured tree traits, including SPGs, were only weakly correlated with diversity and abundance (r<0.15). All correlations reported above are statistically significant (P<0.01).

**Fig 4:**
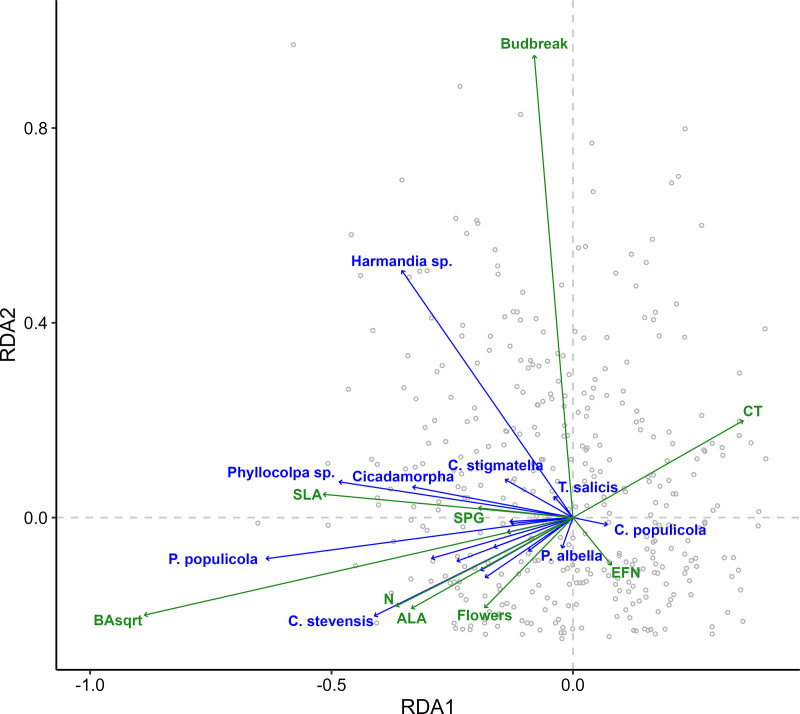
Redundancy analysis for effects of aspen traits (green) on insect community composition (blue). Lengths of trait vectors represent relative values of the corresponding trait and lengths of insect vectors represent relative abundance of the corresponding species. Gray points represent aspen genet averages. RDA1 accounts for 63.0% of the explained variation and 10.7% of the total variation. RDA2 accounts for 17.4% of the explained variation and 3.0% of the total variation. Collectively, the traits explained 21% of the variation. See Table 2 for variance partitioning by trait.

Redundancy analysis (Fig 4) and PERMANOVA (Table 2) revealed that tree size, followed by budbreak timing, SPG concentrations, and CT concentrations, most strongly shaped insect communities overall, accounting for 51, 16, 8, and 8% of the explained variation, respectively. Aspen traits explained an estimated 17.2% of the total variation in insect communities. Size, phenology, and defense chemistry altogether accounted for 83% of that explained variation.

**Table 2:** PERMANOVA results for the effects of aspen traits on insect community composition. 1000 permutations were used. Column descriptions: Source of variation, degrees of freedom, variance explained, F-statistic, P-value.

Source	DF	Var	F	P
BAsqrt	1	0.029	50.112	<0.001
CT	1	0.004	7.516	<0.001
SPG	1	0.004	7.442	<0.001
N	1	0.003	5.019	<0.001
ALA	1	0.003	5.223	<0.001
SLA	1	0.002	3.691	0.001
EFN	1	0.001	1.237	0.229
Flowers	1	0.001	0.986	0.436
Budbreak	1	0.009	15.933	<0.001
Sex	1	0.001	1.088	0.355
Residual	477	0.275		

### Genomic associations and effects on insect communities

In addition to phenotypic effects on insects, we identified genomic associations with community composition, but found no such associations with broader richness, evenness, or diversity metrics. Seventeen SNPs, within 15 unique genes, were associated with insect community composition (Table 3). The functions of these insect-associated genes are largely unknown, though information is available for some gene regions. Three genes were associated with incidence of *E. populella* and appear to be involved in vacuolar functioning in *Arabidopsis* [72], programmed cell death in plants and animals [73–75], and pectin structure in *Populus trichocarpa* [76]. Six genes, involved primarily in cellular transport [77–80], and some of which may be defense-related [81,82], were associated with *P. albella*. Two genes were associated with *Z. scutellaris*, but little is known about their function. Three genes, two of which likely serve regulatory functions [83–86] and one of which is involved in flowering time [87], were associated with *C. stevensis.* One gene of little-known function was associated with *G. septentrionis*. No genomic associations were identified for common insect abundance or diversity (including MDS axes), nor were associations found for functional group abundance or diversity.

**Table 3:** Genomic associations with insects. Column descriptions: common insect species; SNP identifier; gene identifier; short gene description; standardized effect that a minor allele has on the incidence of the insect species when compared to the more dominant allele, as estimated with a logistic mixed effects model of genomic associations; the uncorrected p-value for the association test; and Storey’s positive false discovery rate correction of the p-value.

Insect	SNP	Gene	Annotation	Effect	p-value	q-value
E. populella	Potra001987:15805	Potra001987g15625	vacuolar fusion protein MON1 homolog	−0.493	0.0000047	0.128
E. populella	Potra001987:16306	Potra001987g15625	vacuolar fusion protein MON1 homolog	−0.495	0.0000040	0.128
E. populella	Potra001905:22346	Potra001905g15122	oxysterol-binding family protein	0.517	0.0000021	0.128
E. populella	Potra000664:25828	Potra000664g05124	pectate lyase-like	−0.532	0.0000028	0.128
P. albella	Potra002350:11375	Potra002350g17933	probable voltage-gated potassium channel subunit beta	0.370	0.0000057	0.127
P. albella	Potra003977:65440	Potra003977g23930	probable magnesium transporter NIPA2/NIPA2 isoformX1	−0.373	0.0000014	0.117
P. albella	Potra000432:104584	Potra000432g02304	conserved oligomeric Golgi complex subunit 3-like	0.485	0.0000032	0.117
P. albella	Potra002902:3513	Potra002902g20271; Potra002902g35386	phosphate transporter PH01; Unknown	0.610	0.0000051	0.127
P. albella	Potra002165:8639	Potra002165g16720	delta(7)-sterol-C5(6)-desaturase-like	0.519	0.0000026	0.117
Z. scutellaris	Potra002388:21367	Potra002388g18183	Transmembrane Fragile-X-F-associated protein	0.543	0.0000026	0.140
Z. scutellaris	Potra000678:82497	Potra000678g05264	uncharacterized transporter YBR287W-like	0.435	<0.0000001	<0.001
C. stevensis	Potra003317:9694	Potra003317g21325	protein LATE FLOWERING-like	−0.345	0.0000043	0.129
C. stevensis	Potra003317:9705	Potra003317g21325	protein LATE FLOWERING-like	−0.350	0.0000035	0.129
C. stevensis	Potra000973:118740	Potra000973g08049	SKP1-like protein 21 isoform X1	0.297	0.0000049	0.129
C. stevensis	Potra001290:6891	Potra001290g11127	protein STRUBBELIG-RECEPTOR FAMILY 3 isoform X1	0.401	0.0000058	0.129
C. stevensis	Potra001290:6895	Potra001290g11127	protein STRUBBELIG-RECEPTOR FAMILY 3 isoform X1	0.401	0.0000058	0.129
G. septentrionis	Potra002010:112138	Potra002010g15755	serine/threonine-protein kinase rio1-like	0.496	0.0000010	0.112

In addition to this direct evidence of genomic associations with community composition, we found indirect evidence of links with other insect species. Tukey’s higher criticism tests showed that additional composition-associated genes remain unidentified (Table 3). This meta-test, conducted on p-value distributions from individual genomic association tests of each insect, revealed that five species are likely to have undiscovered associations. Tests for Coleophoridae *sp.*, *Nematus sp.*, *Harmandia sp.*, *Phyllocolpa sp.*, and *C. populicola* contained significantly more p-values below 0.05 than expected if no genomic associations truly existed for those species. Three of the species, *Harmandia sp.*, *Phyllocolpa sp.*, and *C. populicola,* were among the four most heritable (i.e., strong genotypic effects) community members in our population (Fig 1C), which supports the hypothesis that genomic drivers for these species exist.

We also identified genomic associations with tree traits influencing insect communities, providing a second indirect source of evidence for genomic effects on tree extended phenotypes. We found 110 SNPs, within 73 unique genes (Table A in S2 File), associated with three highly heritable aspen traits. Twenty-two SNPs were associated with budbreak timing, one was linked to extrafloral nectary density, and 87 were associated with flower production. These three traits collectively influenced all 17 common herbivores (Fig 3). In the clearest example linking genes to communities through traits, one gene associated with flower production (Potra002010g15755) was also the sole gene associated with *G. septentrionis*, incidence of which was directly linked to flower production (Fig 3).

Comparisons between genomic association models that included tree traits as covariates with those that did not revealed that aspen traits provide the functional bridge between aspen genetics and insects as extended phenotypes. Within our dataset, no SNPs were associated with any insect species once tree trait covariates were added to the genomic models. This result indicates that the detected genomic effects on community composition can be explained entirely through their effects on aspen phenotype.

Gene enrichment analyses identified no significant functional groupings of genes with respect to their influence on aspen phenotypes or extended phenotypes.

## Discussion

This work establishes connections between insect communities of a foundation forest species, the tree traits associated with those insects, and genes affecting those relationships. Our findings support the notion that the genetics of a single species can shape the communities with which it interacts. We found that herbivore communities differ among aspen genotypes and that the composition of those communities is genetically heritable. Further, we found that communities were shaped by heritable aspen traits. We identified 73 genes associated with those community-linked traits and another 15 genes directly associated with the insect communities themselves. Finally, we discovered evidence that additional genes associated with aspen extended phenotypes remain unidentified. These results demonstrate that plant extended phenotypes can be tracked to individual genes [88].

### Aspen-associated insect communities are heritable extended phenotypes

Few studies investigating community heritability exist to date, but they suggest that genetic influences on extended phenotypes are variable across systems and are context dependent. Among community metrics, species richness and community dissimilarity tend to have the highest heritability [88–90]. Our results, and those of Barker et al. [38], differ from those preceding studies in that these metrics were among the *least* heritable of all genetically influenced features measured. These results should not be surprising for our study, however, which was conducted over multiple seasons and years. The factors most affecting insect communities on this time scale may be external to the host plants (e.g., weather patterns, natural enemies, colonization rates). Nonetheless, our findings demonstrate persistent community genetic effects independent of environmental and temporal variation in terms of both insect incidence and, to a lesser extent, insect diversity. These results are consistent with recent findings in *Eucalyptus* [13].

While community genetics studies often evaluate the effects of intraspecific variation on the numbers of species and individuals occurring in communities, they rarely consider effects on *which* species occur. The incidence of many species was highly heritable for this aspen population – more so than heritability of some tree traits. Although overall insect community diversity differs only weakly with genetic variation in aspen, the members comprising those communities are more strongly affected. The conditions that influence abundance are not necessarily the same as those affecting incidence. For example, a plant’s chemical profile may determine its suitability as a host for a particular species but, within the acceptable chemical range, other factors may be more important drivers of abundance.

Due to the limitations of binomial models, confidence intervals for heritability of insect incidence were much wider than for quantitative tree traits and diversity metrics (i.e., continuous response variables). This means that there is a greater uncertainty in the exact degree of heritability for insect incidence than for insect diversity and tree traits. Even if we employed the more conservative approach of using the lower 95% confidence bound of heritability instead of the mean estimate, insect incidence would remain much more heritable on average than insect diversity and abundance and only slightly below the heritability of tree traits.

The most heritable insect species were specialist leaf-modifiers. Our finding that occurrence of the leaf-galling fly *Harmandia sp.* was highly heritable confirms results of Bernhardsson et al. [89], who found that galling insects were similarly heritable on *Populus tremula*, and Simon et al. [91], who found the same for *Harmandia* on *Populus trichocarpa*. Furthermore, *Harmandia* were strongly associated with budbreak phenology, a phenotype that was also highly heritable and associated with aspen genes, indicating an indirect genomic association. Free-feeding herbivores, which are less intimately associated with their host, were much less heritable. The one strong genotypic association with free-feeding herbivores occurred with the aphid *Chaitophorus stevensis.* This result corroborates other similar findings for aphids [38,92]. In general, results from this and related studies suggest that heritability of community phenotypes is correlated with the degree of specialization and physical intimacy of insect-plant relationships.

### Heritable aspen traits are linked to insect communities

Across ecosystems, plant trait expression plays a major role in shaping communities, and herbivores are particularly responsive to it [93]. These ecologically relevant traits are often heritable. This study, along with previous work in the system [26,37,38], clearly links community diversity and structure to genetically-mediated tree traits.

In our aspen population, the plant trait most strongly associated with insect communities was, unsurprisingly, tree size. Larger trees supported more densely populated and diverse communities. These results accord with the trend of larger trees providing more habitat for associated organisms [94,95]. One outlier of this pattern was the aphid *C. populicola*, which was observed *less* frequently on larger trees – likely due to its penchant for feeding on new shoots in the upper canopy [96] beyond our sampling area.

Foliar secondary chemistry was also associated with community composition and diversity, albeit counter-intuitively. Salicinoid phenolic glycosides (SPGs) are known to have detrimental impacts on herbivores, especially lepidopterans [29,97], but all species except one were positively associated with SPGs here. The affected insects were almost entirely specialists, which are likely adapted to SPGs. For example, *Chrysomela crotchi* metabolizes SPGs to produce salicylaldehydes for their own defense [98] and gregarious sawflies like *Nematus spp.* exhibit comparable defense co-opting strategies [99–101]. Similarly, condensed tannins (CTs) had positive impacts on half of the species they affected. This evidence, combined with the decreased taxonomic diversity associated with CTs, indicates that the few insect species present on trees with high CT concentrations are particularly adapted to these compounds. In short, the common members of aspen-associated herbivore communities appear to be well-adapted to its chemical defenses, in contrast to less common herbivores and generalists [29]. Aspen defense compounds are likely more important in determining which species do *not* occur on trees, as opposed to differences in incidence rates of the (primarily specialist) species that do occur.

Physical and nutritional leaf traits are also associated with herbivore damage and abundance [102–104] as well as community composition [105]. Our results indicate similarly: leaf thickness and nitrogen concentration were associated with herbivore richness, but to a lesser degree than tree size and defense chemistry.

Ontogenetic trajectories play another important role in shaping extended phenotype communities. Assemblages of common insects in this study were quite different from those observed in the garden two years earlier [38]. These differences are likely partially attributable to age-related (4–5 vs. 6–7 years old) changes in key aspen traits. In this later study, trees were much larger and had begun to reproduce, the canopy had closed, and foliage had lower secondary metabolite and higher nitrogen concentrations [26]. These ontogenetic shifts expose herbivores to different tree phenotypes and environmental conditions (e.g., temperature, natural enemies), such that community composition changes over time.

This research focused on the bottom-up effects of plants on insect communities, an admittedly limited approach. Natural enemies such as predators and parasitoids, for example, can strongly shape insect communities [106,107]. Because top-down drivers are generally stronger than bottom-up factors for chewing, sucking, and gall-forming insects [108], they may have partially obscured the effects of plant phenotypic variation in our study.

Top-down effects on insect herbivores may themselves be influenced by plant intraspecific variation. For example, plant volatiles (not measured here) may augment protection for *Populus* [109,110] and vary by genet [111]. Extrafloral nectaries are also expected to decrease herbivore abundance by attracting natural enemies [112–114], but were not associated with the only common natural enemy in our study, ants. Nectaries did negatively influence incidence of *Cicadamorpha spp.,* Coccoidea *sp.,* and *P. populicola*. It is plausible that these impacts were mediated by unquantified natural enemies.

As evidenced by the relatively low overall explanatory power – 21% as determined via redundancy analysis – of aspen phenotype on insect community variability, external factors clearly play a role. Insect population dynamics, community interactions, predator-prey dynamics, migration, and abiotic environmental factors all contribute to community dynamics. Gossner et al. [21], for example, found that spatial and climatic factors driving arthropod communities far outweighed the effects of intraspecific variation in an oak system. That 21% of variation in a generally highly mobile insect community can be attributed to host plant phenotype is a significant finding. Whether these patterns would hold across environmental gradients remains to be seen [21].

### Aspen genes contribute to shaping insect communities

Genotypic effects of plants on associated insects are common, but few studies have identified candidate genes associated with community phenotypes. *Populus* genes, specifically, have been associated with insect herbivore metrics, including damage rates [115,116] and community diversity [89]. The 15 candidate genes identified by this study do not overlap with genes previously linked to aspen traits or community phenotypes [36,37], likely due to our use of an improved single-marker association model and the aforementioned ontogenetic shifts. While the functions of these 15 genes remain poorly understood, what is known aligns well with their putative ecological relevance.

This work provides strong evidence that aspen genes have influences beyond the immediate phenotypes those genes control. The most compelling direct evidence relates to the poplar petiole gall moth, *Ectoedemia populella*. We identified three genes associated with this species. The first, Potra000664g05124, has been linked to enzymes involved in structuring pectin in cell walls and is important for wood formation and vascular tissue in *Populus trichocarpa* [76]. Galling insects like *E. populella* depend upon, and often manipulate, the morphology and physiology of their host [117,118]. These processes are likely influenced by plant structural enzymes. Potra000664g05124 has been similarly linked to parasitic root-knot nematodes [119], which interact with hosts in a manner mechanistically similar to *E. populella* [120]. In Arabidopsis, the gene homologous to Potra000664g05124 has also been linked to disease immunity [121]. The second gene associated with *E. populella* in our study, Potra001987g15625, is involved with vacuolar trafficking, biogenesis, and plant growth [72] as well as programmed cell death in *Arabidopsis* [73]. The third gene, Ptra001905:g15122, also has links to programmed cell death [74,75]. Like most galling species, *E. populella* relies on the host’s nutrient transport systems and photosynthetic products for sustenance. Programmed cell death is linked to oxidative stress in plants [122–124] and, unsurprisingly, *E. populella* has been shown to manipulate oxidative stress responses in *Populus* [125]. Another compelling genomic association exists for the moth *Gluphisia septentrionis*. The aspen gene (Potra002010g15755) linked to *G. septentrionis* was also linked to flower abundance, the tree trait most correlated with abundance of this species. Why plant reproduction, and its associated genes would impact a larval lepidopteran herbivore is unclear, but may be related to tradeoffs among growth, defense, and reproduction [26]. Insufficient information exists about the other insect-associated candidate genes to evaluate their mechanisms but should be considered for further investigation.

Genomic association models are continually improving in their ability to detect genetic links beyond the systems for which they were developed, but further innovation is needed for their application to extended phenotypes. The genomic association model used here was selected over the more commonly used approach because it better accounts for the genetic, phenotypic, and environmental correlation structure inherent in the system. This method is more conservative but has statistical properties that allow us to be quite confident that the genes identified as being associated with extended phenotypes truly are, even with the slightly lenient false detection threshold of 15%. Still, the results from Tukey’s higher criticism test indicate that additional genetic drivers of extended phenotypes remain undetected in our system. These tests revealed for five insect species that, among all genetic association tests, p-values trended lower than expected by random chance. This situation arises when a real effect exists diffusely across many effectors [55]. Even heritability was not a reliable predictor of genomic effects. No specific gene associations were identified for three of the four *most* heritable herbivore species, while genetic links were identified for moderately heritable species. These undetected effects are likely smaller than the model had power to detect and/or are driven by rare gene variants, which our model evaluation proved are difficult to detect. Both the patterns of missing heritability and our inability to identify genes whose effects are clearly present are consistent with a highly polygenic architecture in aspen, in which expression of key functional traits is determined by many genes of low effect [37]. These features also demonstrate why new system-specific methodologies are needed.

In summary, our research incorporated results collected over several years for 1,568 trees, 113,674 SNPs, 13 phenotypic traits, 46 extended phenotypes, and nearly 650,000 individual insects. This extensive effort revealed scores of genes associated with particular tree traits, which in turn were linked to insect communities, as well as 15 candidate genes whose phenotypic mediators of insect assemblages remain undiscovered. By identifying genomic links between intraspecific variation and herbivores, and the traits mediating those interactions, this work exemplifies the value of innovation in community genetics research. New, more powerful genomic association tools are needed, however, when the genetic determinants of plant traits and associated communities are many genes of low effect.

## Supporting information

S1 File Details of the Genomic Association Analysis Methods. (DOCX)

S2 File Supplemental Tables, Figures, and Captions. (DOCX)
