# Supplementary material for: Canopy insect communities are shaped by the genes and phenotypes of their aspen hosts
Source: PLoS One. 2025 Jul 17;20(7):e0327554. doi: 10.1371/journal.pone.0327554 (PMC12270107; doi:10.1371/journal.pone.0327554)
Supplement: S1 File — (DOCX) [file pone.0327554.s001.docx]

**Supplemental Document 1:**

**Genomic association model comparison**

**Summary**

To identify an appropriate model for detecting genomic associations with extended phenotypes in our aspen system, we fit four models to simulated aspen populations and compared their statistical properties. These models include three competing mixed effects models in addition to the model used in our previous work (Barker et al. 2019; Riehl et al. 2023). The artificial aspen populations were simulated using properties similar to those present in a natural common garden population of aspen in order to be as representative of our real-world system as possible.

**Simulations**

Aspen populations with 600 individuals each were simulated. All populations possessed four intercorrelated phenotypic traits, clonal population structure, temporal variation, genomic variation, and an associated extended phenotype. Traits were modelled after the ecologically relevant *Populus* traits: tree size, specific leaf area, condensed tannin concentration, and phenolic glycoside concentration. The extended phenotypes were affected by clonal, phenotypic, and genomic variation of the plants as well as by random environmental variation. We modelled these extended phenotypes to 1) match how insect herbivores are affected by the natural *P. tremuloides* population, 2) respond to host genotype and traits, and 3) exhibit temporal variation. Simulated populations contained 200 genets each with three replicate trees per genet. Because the reference population of *P. tremuloides* does not have significant population structure (Barker et al. 2019), genets were independent and identically distributed (*i.i.d.*) in these simulations. We projected traits and extended phenotypes over four time periods, the same number of time periods as surveyed in the reference system.

We introduced genetic variation, at a molecular level, by generating an ecologically relevant SNP within the population. For the purposes of this simulation study, the SNP was assumed to be independent from any other genetic effects encompassed by clonal variation. This corresponds to the underlying assumptions of the genomic association models being tested. We encoded a SNP value for each genet as -1 for homozygous major variants (AA), 0 for heterozygous variants (Aa), or 1 for homozygous minor variants (aa). This encoding allowed us to test the additive effects of minor alleles. We assigned SNP variants to each genet by drawing randomly from the set {-1, 0, 1} with probabilities $p_{AA}$, $p_{Aa}$, and $p_{aa}$, respectively. As such, the SNP variable $Z$ follows a multinomial distribution for each genet $g$: $Z_{g}\sim\text{Multinom}\left( p_{AA}, p_{Aa}, p_{aa};n=1 \right)$. We assigned variant probabilities by specifying the minor allele frequency $q$ of the population in each simulation and assuming Hardy-Weinberg equilibrium:$p_{AA}={(1-q)}^{2}$; $p_{Aa}=2q(1-q)$; and $p_{aa}=q^{2}$ (Weinberg 1908; Hardy 2010). The SNP was simulated with either low minor allele frequency $q=0.1$ or high minor allele frequency $q= 0.4$. Both selected minor allele frequencies regularly occurred among SNPs in the reference *P. tremuloides* population (Lind-Riehl et al. *in review*) where the median minor allele frequency was 0.2.

| $X_{i}\left( t \right)=Z\phi_{i}+{G\gamma}_{xi}+\left( t-1 \right)\rho_{i}\boldsymbol{1}+\delta_{xi}\left( t \right)+\varepsilon_{xi}(t)$ | (1) |
| --- | --- |

We then simulated four genetically mediated and intercorrelated tree traits with the model given by equation 1 above. Here, $X_{i}(t)$ lists the values of a trait ($i$) at a given time point ($t$) for all 600 individuals. $Z$ lists the values of the SNP genotype for each individual and $\phi_{i}$ is a coefficient describing the fixed SNP effect on the trait. The systemic genet-specific effects on the trait are given by $G\gamma_{xi}$. The term $\gamma_{xi}$ contains the 200 random effects, one for each genet. $G$ is a genet membership matrix, indicating if an observation belongs to a genet. The multiplication of $G\times\gamma_{xi}$ assigns the correct genet-specific effect to each member of that genet. Each trait grows at a constant rate between each time step, as determined by the growth coefficient $\rho_{i}$. The vector of ones ($\boldsymbol{1}$ in equation 1) indicates that the growth coefficient is the same for all individuals in a simulation, for a given trait. Temporal autocorrelation is generated by the first order autoregressive process $\delta_{xi}\left( t \right)=\delta_{xi}\left( t-1 \right)+G\nu_{xi}\left( t \right)$, wherein $G\nu_{xi}\left( t \right)$ are time-dependent genet effects (i.e., the random interaction between genet and time). Here, $\nu_{xi}\left( t \right)$ is contains the 200 genet effects for the trait at the given time$.$ The time-dependent genet effects are independent among all pairs of time points for each trait. Again, $G$ indicates genet identity. $\delta_{xi}\left( t \right)$, then, represents the accumulation of time-dependent genet effects at each time step for each individual. The residual variance for trait $i$ at a given time is denoted $\varepsilon_{xi}\left( t \right)$. Residual variation is intercorrelated among the four traits according to the covariance matrix $\Sigma_{x}$ (for any two traits $j$ and $k$, $\Sigma_{x}=cov\left( \varepsilon_{xj}\left( t \right), \varepsilon_{xk}\left( t \right) \right)$) across time and individuals. All random effects, and the error terms are normally distributed and independent, with variance determining effect magnitudes. With this model formulation, phenotypic expression of intercorrelated traits is determined by fixed and random genetic effects, fixed temporal effects, temporally autocorrelated interactions of random clonal and temporal effects, and random residual effects.

To ensure simulation of realistic phenotypes, we chose parameter values derived from the natural aspen population (Lind-Riehl et al.; Barker et al. 2018, 2019; Cole et al. 2021). For all simulations, we set the systemic genet-specific variance ($\sigma_{xg}^{2}$) equal to 0.5 and the time-specific variance ($\sigma_{x\nu}^{2}$) equal to 0.2, which match typical relative magnitudes of genetic and temporal variance of *P. tremuloides* traits, respectively. The trait growth coefficients were set to match annual trends in four ecologically important tree traits: tree size, specific leaf area, condensed tannin concentration, and phenolic glycoside concentration, respectively ($\rho=[0.87, 0.68, 0.12, 0.02]$). Covariance among the residuals of these traits ($\Sigma_{x}$) was also taken from estimates of the natural population (Table B) and was constant among all simulated populations. We simulated three SNP conditions: no effect, small effect, or moderate effect, on the first trait only ($\phi= \left[ 0, 0, 0, 0 \right],$ $\phi=[0.1, 0, 0, 0]$, or $\phi=[0.2, 0, 0, 0]$, respectively). This allows us to compare true associations with indirect effects mediated by the intercorrelations among traits.

| $Y(t)=\boldsymbol{X}(t)\alpha+Z\beta+{G\gamma}_{y}+\tau_{y}(t)\boldsymbol{1}+{G\nu}_{y}(t)+\varepsilon_{y}(t)$ | (2) |
| --- | --- |

We also simulated an extended phenotype for each population, with varying degrees of dependence on population characteristics and environmental factors with the model given by equation 2 above. Here, $Y\left( t \right)$ are the extended phenotype values for all individuals at time $t$. $\boldsymbol{X}\left( t \right)$ is a phenotype matrix containing trait values for all individuals at time *t.* $\boldsymbol{X}\left( t \right)$ has four columns corresponding to each of the four traits generated with equation 1. $\alpha$ lists the four additive effects of each trait on the extended phenotype. $Z$ are the SNP values for each individual, as in equation 1, and $\beta$ is a fixed coefficient of the direct SNP effect on the extended phenotype. Systemic genet-specific effects on the extended phenotype are given by $G\gamma_{y}$. The term $\gamma_{y}$ contains the 200 random genet effects and $G$ is the genet identity matrix described previously. Random temporal effects, one for each time step, are given by $\tau_{y}\left( t \right)$. A random interaction between genet and time is given by $G\nu_{y}\left( t \right)$. The term $\nu_{y}\left( t \right)$ contains 200 random genet effect values. Residual variation for the extended phenotype is given by $\varepsilon_{y}\left( t \right)$. All random effects and the residual error are normally distributed, independent, and effect magnitudes are determined by variance components. With this formulation, expression of the extended phenotype is determined by fixed trait effects, fixed and random genetic effects, random effects of the interaction between genetic and temporal factors, and random residual variation.

In each simulation, we set the effect of trait 1 on the extended phenotype$\left( \alpha_{1} \right)$ to 0.00, 0.125, or 0.250; and we set the effect of the other traits ($\alpha_{2}$, $\alpha_{3}$, $\alpha_{4}$) to either 0.000 or 0.250 independently from each other and from $\alpha_{1}$. With different combinations of $\alpha$ values, many possible effects of phenotype on extended phenotype were present in our simulations. We also varied the SNP effects across simulations by setting $\beta$ to one of 0.00, 0.125, 0.250, or 0.500. For all simulations, we set the clonal variance for the extended phenotype ($\sigma_{yg}^{2}$) to 0.55 and the temporal variance ($\sigma_{y\tau}^{2}$) to 0.79. These values, and the residual variation $\sigma_{y}^{2}=0.64$, were typical among estimates of extended phenotype effects in a natural population of *P. tremuloides*. The interaction effect was varied by setting $\sigma_{y\nu}^{2}$ equal to one of 0, 0.25, or 0.50. We simulated populations, SNPs, and extended phenotypes together, under each combination of parameters in the extended phenotype model, each value of $\phi_{i}$ from the trait model component, and each minor allele frequency $q$ considered. 3,000 simulations each were conducted under cases where $\alpha_{2}=\alpha_{3}=\alpha_{4}=0$ and 2,000 simulations were conducted for all other cases. For cases in which any of $\alpha_{2}$, $\alpha_{3}$, or $\alpha_{4}$ were greater than 0.250, we simulated under limited conditions: $\phi_{1}$was restricted to either 0 or 0.2, $\sigma_{y\nu}^{2}$ was restricted to either 0 or 0.5, and $q$ was restricted to 0.1 only. This was done to reduce complexity and computation effort of the simulation study. In total, we simulated 450,000 populations under one of 180 parameter combinations.

Association analyses

To these simulated data, we fit a series of competing models and assessed their ability to detect genetic effects on an extended phenotype and their statistical properties. We evaluated and compared four separate genomic association models for each simulated data set. We first fit three types of mixed effects models, each with different random effects specification. Two versions of these mixed models were fit: one including traits as covariates and one not including trait covariates. The final model, against which the others were compared, regressed the best linear unbiased predictor (BLUP) of genetic effects against the genomic marker.

In general, the fitted mixed models follow from equation 2, but with slight differences in how the effects of genet, time, and their interaction were specified. The first mixed model MM_add_ (eq. 3) is the full additive model and exactly matches the extended phenotype simulation model (eq. 2), in which there are fixed SNP effects $Z\beta$, random clonal effects $G\gamma_{y}$, random temporal effects $\tau_{y}\left( t \right)\boldsymbol{1}$, random interactions between genet and time $G\nu_{y}(t)$, and fixed trait effects $X\left( t \right)\alpha$ in the versions of the model that include trait covariates. The next two models MM_nestT_ (eq. 4) and MM_nestG_ (eq. 5) are nested random effects models. MM_nestT_ assumes systemic random temporal effects and that genet random effects are entirely time-specific. Conversely, MM_nestG_ assumes systemic random genet effects and that temporal random effects are entirely genet-specific. The goal for all fitted mixed models is to estimate the effect of the causal SNP on the extended phenotype ($\hat{\beta}$), independent of the other terms. The estimate of $\hat{\beta}$ is conditional upon the random effects: specifically, on the overall covariance structure constructed from covariance parameters $\theta$. These covariance parameters are estimated via restricted maximum likelihood and then the fixed effects parameters (including $\hat{\beta}$) are estimated using maximum likelihood, conditional on the estimated $\hat{\theta}$. Finally, the random effects are predicted (as best linear unbiased predictors: BLUPs) from the covariance structure, fixed effects, and observations $Y\left( t \right).$ The term in the mixed model equation $\left[ X\left( t \right)\alpha\right]$ indicates that a model was fit either with or without trait covariates.

| **Fitted Mixed Effects Models** | | | |
| --- | --- | --- | --- |
| **Name** | **R formula** | **Equation** | **Eq. #** |
| MM_add_ | “Y ~ [X] + Z + (1\|genet) + (1\|time) + (1\|time:genet)” | $Y\left( t \right)=\left[ X\left( t \right)\alpha\right]+Z\beta+G\gamma_{y}+\tau_{y}\left( t \right)\boldsymbol{1}+G\nu_{y}\left( t \right)+ \varepsilon_{y}(t)$ | (3) |
| MM_nestT_ | “Y ~ [X] + Z + (1\|time) + (1\|time:genet)” | $Y\left( t \right)=\left[ X\left( t \right)\alpha\right]+Z\beta+\tau_{y}\left( t \right)\boldsymbol{1+}G\nu_{y}(t)+\varepsilon_{y}(t)$ | (4) |
| MM_nestG_ | “Y ~ [X] + Z + (1\|genet) + (1\|time:genet)” | $Y(t)=\left[ X\left( t \right)\alpha\right]+Z\beta+G\gamma_{y}+G\nu_{y}(t)+\varepsilon_{y}(t)$ | (5) |

The final model, against which these mixed-effects models were compared, named G_BLUP_ (eq. 6), has been used by our group previously to detect genomic effects and identify candidate genes (Barker et al. 2019; Riehl et al. 2023). This model regresses the BLUP of the clonal random effect on the SNP value of each genet $Z_{g}$. Here, the clonal BLUP $\hat{\gamma}_{y}$ is first extracted from the mixed model $Y\left( t \right)=G\gamma_{y}+\tau_{y}\left( t \right)\boldsymbol{1}+\varepsilon(t)$ that does not include the SNP. In this way, $\hat{\gamma}_{y}$ represents the predicted *overall* genetic effect on the extended phenotype. Then, equation 6 correlates that effect with the SNP.

| G_BLUP_ | $\hat{\gamma}_{y}=Z_{g}\beta+\varepsilon_{g}$ | (6) |
| --- | --- | --- |

An important assumption underlying the G_BLUP_ model is that if a SNP is associated with the extended phenotype, then it should also be associated with the overall genetic effect ($\gamma$). This type of two-step approach was described by Stich et al. (2008) and is relatively common (e.g., Sikorska et al. 2013, 2015; Chhetri et al. 2019; Esmaeili-Fard et al. 2021). The two-step BLUP approach is used instead of full mixed effects models for its computational efficiency. In a system with many SNPs, a mixed model approach can be computationally expensive because of the need to estimate covariance parameters ($\theta)$ and invert the resulting covariance matrix (i.e., $\Sigma_{\theta}$) for each SNP. To address this problem, the G_BLUP_ method fits a single covariance matrix for each response variable and recycles the resulting BLUP values, rather than fitting the covariance matrix for each SNP$\times$response combination.

Model Comparison

We evaluated the fit of all models, for all simulated populations, by comparing the estimated value of $\hat{\beta}$ to the true genetic effects on the extended phenotype and by the proportion of tests indicating that the null hypothesis ($H_{0}: \beta=0$) was true (i.e., the rejection rate). We assessed the accuracy of each model under the various conditions (i.e., parameter space). All simulations and model fitting were conducted using the R statistical software library (R Core Team 2021). The G_BLUP_ model was fit using the “lm” function from the R package “stats” and mixed effects models were fit using the “lme4” package (Bates et al. 2015). Statistical tests of $\hat{\beta}$ for mixed models were conducted with the “lmerTest” package (Kuznetsova et al. 2017) , and we used Satterthwaite degrees of freedom (Fai and Cornelius 1996). We also calculated the 2.5% and 97.5% quantiles for estimates of $\hat{\beta}$, which show the values between which  $\hat{\beta}$ was estimated for 95% of the simulations.

Direct, indirect, and total SNP effects

In these simulated systems, the extended phenotype is affected by the causal SNP in two ways: directly and indirectly through the traits. The “direct” effect of the SNP on $Y(t)$ is simply $\beta$. While in real-world systems there can be no *direct* effect of the genetics of one species on another (genes cannot encode for other organisms), this can be interpreted as the cumulative indirect genetic effects on an extended phenotype that are mediated by all traits other than the ones included in **X**. The indirect SNP effect is dependent upon both the SNP effect on the measured traits (the column vector $\phi$) and the effect of these traits on the extended phenotype (the column vector $\alpha$). In fact, the indirect SNP effect is the product $\phi^{'}\alpha$. The total SNP effect on the extended phenotype is then equal to $\beta+\phi^{'}\alpha$. This is proven algebraically by substituting the right-hand side of equation 1 in for $\boldsymbol{X}(t)$ in equation 2 (i.e., eq. 9).

| $Y\left( t \right)=X\left( t \right)\alpha+Z\beta+\ldots$  $=(Z\phi^{'}+\ldots)\alpha+ Z\beta+\ldots$  $=Z\left( \beta+\phi^{'}\alpha\right)+\alpha\left( \ldots\right)+\ldots$ | (9) |
| --- | --- |

**Comparison Results**

All the models considered were reasonably accurate at estimating SNP effects on extended phenotype on average before including trait covariates, but the mixed models were an improvement over the G_BLUP_ method. All models had a slight tendency to underestimate the effect size, but the G_BLUP_ was the most biased (Table C). All the models had comparable precision, as indicated by the empirical distribution of the estimated SNP effect (see 2.5% and 97.5% quantiles in Table C). All estimates of SNP effects were identical among the mixed models for each population.

Whereas the models estimated effect sizes similarly, they did not determine significance of these effects at comparable rates. Both the G_BLUP_ and MM_nestT_ models had extremely high Type I error rates (34.3% and 25.0%, respectively), rendering them invalid for testing statistical hypotheses (Figure E). The remaining mixed effects models MM_full_, MM_nestG_ both had well-controlled Type I error rates (5.7% and 5.5%, respectively) and power to detect even the smallest effects ($\beta=0.125$) around 35% of the time on average (Table D). Because of their inappropriately high Type I error rates, both G_BLUP_ and MM_nestT_ were removed from consideration as genomic association models for this system.

Adding trait covariates to the remaining genomic association models resulted in similar patterns as without trait covariates. The models containing trait covariates were biased slightly toward zero in their estimation of SNP effects relative to models without covariates (Table E). This is because these versions of the models estimate the *direct* SNP effect, which is smaller than the *total* SNP effect estimated by the models without covariates. Both versions of each model are estimating the effects accurately but are estimating *different* effects (i.e., direct vs. total). The ability to detect significant associations was also slightly reduced when trait covariates were included (Table F) for the same reason.

In addition to the expected impact that the true value of $\beta$ (SNP effect on extended phenotype) had on the detection of significant associations (Figure E), the parameters $\alpha$ (direct trait effect on extended phenotype), $\phi$ (direct SNP effects on traits), and $q$ (minor allele frequency), all substantially impacted the estimation of $\hat{\beta}$ (total estimated SNP effect on extended phenotype) and rejection of the null hypothesis (Figures F, G, and H). Increasing the effect of a SNP on trait 1 and the effect of trait 1 on the extended phenotype had the largest impacts. All else equal, increasing the indirect SNP effect ($\phi^{'}\alpha$) increased the ability of the models to detect significant effects (Figure F). This is unsurprising, given that the total SNP effect is increased, and the model is designed to detect those effects. The second largest driver of detection probability was the minor allele frequency $q$. All else equal, the power of our mixed models to detect SNP effects was greatly increased among SNPs with 40% minor allele frequencies when compared to SNPs with 10% minor allele frequency (Figure G). This has important implications for real-world application; genomic effects will be more difficult to detect among rare gene variants. In addition to the indirect genetic impact that trait 1 had on detecting association, the remaining trait effects also impacted detection rate. Increasing the effects of traits 2, 3, or 4 ($\alpha_{2}$, $\alpha_{3}$, or $\alpha_{4}$) on the extended phenotype decreased the ability to detect significant SNPs (Figure H) and traits that were more strongly correlated with trait 1 had larger impacts. Increasing the random interaction effects of genet and time had only minor effects on detection (Figure I). Accounting for traits as covariates in the models eliminated these detrimental impacts (Figure J, and K), demonstrating that utilizing both versions of the model allows users to infer whether the traits included as covariates are mediating the relationships between genes and extended phenotypes, or if other, unmeasured traits are responsible.

**Supplemental References**

Barker HL, Holeski LM, Lindroth RL (2018) Genotypic variation in plant traits shapes herbivorous insect and ant communities on a foundation tree species. PLoS One 13:1–21. https://doi.org/10.1371/journal.pone.0200954

Barker HL, Riehl JF, Bernhardsson C, et al (2019) Linking plant genes to insect communities: Identifying the genetic bases of plant traits and community composition. Mol Ecol 28:4404–4421. https://doi.org/10.1111/mec.15158

Bates D, Mächler M, Bolker BM, Walker SC (2015) Fitting linear mixed-effects models using lme4. J Stat Softw 67:. https://doi.org/10.18637/jss.v067.i01

Chhetri HB, Macaya-Sanz D, Kainer D, et al (2019) Multitrait genome-wide association analysis of Populus trichocarpa identifies key polymorphisms controlling morphological and physiological traits. New Phytol 223:293–309. https://doi.org/10.1111/nph.15777

Cole CT, Morrow CJ, Barker HL, et al (2021) Growing up aspen: Ontogeny and trade-offs shape growth, defence and reproduction in a foundation species. Ann Bot 127:505–517. https://doi.org/10.1093/aob/mcaa070

Esmaeili-Fard SM, Gholizadeh M, Hafezian SH, Abdollahi-Arpanahi R (2021) Genome-wide association study and pathway analysis identify NTRK2 as a novel candidate gene for litter size in sheep. PLoS One 16:1–16. https://doi.org/10.1371/journal.pone.0244408

Fai AH-T, Cornelius PL (1996) Approximate F-tests of multiple degree of freedom hypotheses in generalized least squares analyses of unbalanced split-plot experiments. J Stat Comput Simul 54:363–378. https://doi.org/10.1080/00949659608811740

Hardy AGH (2010) Mendelian Proportions in a Mixed Population Published by : American Association for the Advancement of Science Stable URL : http://www.jstor.org/stable/1636004. Adv Sci 28:49–50

Kuznetsova A, Brockhoff PB, Christensen RHB (2017) lmerTest Package: Tests in Linear Mixed Effects Models. J Stat Softw 82:1–26. https://doi.org/10.18637/JSS.V082.I13

Lind-Riehl J, Cole CT, Morrow CJ, et al Genomic and transcriptomic analyses reveal polygenic architecture for ecologically-important functional traits in aspen (Populus tremuloides Michx.)

R Core Team (2021) R: a language and environment for statistical computing

Riehl JFLL, Cole CT, Morrow CJ, et al (2023) Genomic and transcriptomic analyses reveal polygenic architecture for ecologically important traits in aspen (Populus tremuloides Michx.). Ecol Evol 13:1–23. https://doi.org/10.1002/ece3.10541

Sikorska K, Montazeri NM, Uitterlinden A, et al (2015) GWAS with longitudinal phenotypes: Performance of approximate procedures. Eur J Hum Genet 23:1384–1391. https://doi.org/10.1038/ejhg.2015.1

Sikorska K, Rivadeneira F, Groenen PJF, et al (2013) Fast linear mixed model computations for genome-wide association studies with longitudinal data. Stat Med 32:165–180. https://doi.org/10.1002/sim.5517

Stich B, Möhring J, Piepho HP, et al (2008) Comparison of mixed-model approaches for association mapping. Genetics 178:1745–1754. https://doi.org/10.1534/genetics.107.079707

Weinberg W (1908) Über den nachweis der vererbung beim menschen

**Supplemental Tables and Figures**

Tables

Table B: *Covariance among simulated traits (*$\Sigma_{x}$*). These covariances are derived from covariance structure among basal area (*$X_{1}$*), specific leaf area (*$X_{2}$*), phenolic glycoside concentration (*$X_{3}$*), and condensed tannin concentration (*$X_{4}$*) of a real aspen population.*

|  | $\boldsymbol{X}_{\boldsymbol{1}}$ | $\boldsymbol{X}_{\boldsymbol{2}}$ | $\boldsymbol{X}_{\boldsymbol{3}}$ | $\boldsymbol{X}_{\boldsymbol{4}}$ |
| --- | --- | --- | --- | --- |
| $\boldsymbol{X}_{\boldsymbol{1}}$ | 0.5100 | 0.1938 | -0.0714 | -0.1683 |
| $\boldsymbol{X}_{\boldsymbol{2}}$ | 0.1938 | 0.5100 | -0.0663 | -0.1581 |
| $\boldsymbol{X}_{\boldsymbol{3}}$ | -0.0714 | 0.0663 | 0.5100 | -0.1479 |
| $\boldsymbol{X}_{\boldsymbol{4}}$ | -0.1683 | -0.1581 | -0.1479 | 0.5100 |

Table C: *Mean estimates of SNP effects*$\hat{\beta}$ *for each true value of* $\beta$ *for models without trait covariates. These results are averaged across all levels of* $\alpha$*,* $\phi$*, and* $q$ *for a total of* $N=90000$ *simulations per model. The 2.5% and 97.5% percentiles of the empirical distribution of* $\hat{\beta}$ *are given in parentheses.*

|  | $\boldsymbol{\beta=0}$ | $\boldsymbol{\beta=0.125}$ | $\boldsymbol{\beta=0.25}$ | $\boldsymbol{\beta=0.375}$ | $\boldsymbol{\beta=0.5}$ |
| --- | --- | --- | --- | --- | --- |
| **G_BLUP_** | 0.009 (-0.142, 0.162) | 0.107 (-0.045, 0.266) | 0.203 (0.049, 0.37) | 0.3 (0.139, 0.477) | 0.395 (0.222, 0.581) |
| **MM_add_** | 0.011 (-0.16, 0.182) | 0.12 (-0.05, 0.298) | 0.227 (0.055, 0.412) | 0.333 (0.157, 0.527) | 0.436 (0.249, 0.637) |
| **MM_nestG_** | 0.011 (-0.16, 0.182) | 0.12 (-0.05, 0.298) | 0.227 (0.055, 0.412) | 0.333 (0.157, 0.527) | 0.436 (0.249, 0.637) |
| **MM_nestT_** | 0.011 (-0.16, 0.182) | 0.12 (-0.05, 0.298) | 0.227 (0.055, 0.412) | 0.333 (0.157, 0.527) | 0.436 (0.249, 0.637) |

Table D: Mean estimates of SNP effects ($\hat{\beta}$) for each true value of $\beta$ for mixed models with included trait covariates. These results are averaged across all levels of $\alpha$, $\phi$, and $q$ for a total of $N=90000$ simulations per model. The 2.5% and 97.5% percentiles of $\hat{\beta}$ are given in parentheses. The G_BLUP_ model was not included in this comparison due to the logistical challenges associated with adding covariates to this model.

|  | $\boldsymbol{\beta=0}$ | $\boldsymbol{\beta=0.125}$ | $\boldsymbol{\beta=0.25}$ | $\boldsymbol{\beta=0.375}$ | $\boldsymbol{\beta=0.5}$ |
| --- | --- | --- | --- | --- | --- |
| **MM_add_(X)** | 0 (-0.163, 0.162) | 0.109 (-0.052, 0.278) | 0.217 (0.053, 0.392) | 0.323 (0.155, 0.508) | 0.426 (0.246, 0.621) |
| **MM_nestG_(X)** | 0 (-0.165, 0.168) | 0.11 (-0.055, 0.284) | 0.217 (0.049, 0.398) | 0.323 (0.15, 0.514) | 0.426 (0.242, 0.628) |
| **MM_nestT_(X)** | 0 (-0.164, 0.162) | 0.109 (-0.052, 0.278) | 0.217 (0.053, 0.392) | 0.323 (0.155, 0.508) | 0.426 (0.246, 0.621) |

Table E: *Rejection rate corresponding to the null hypothesis* $H_{0}:\beta=0$ *for two mixed models with included trait covariates. The rejection rates are averaged across all levels of* $\alpha$*,* $\phi$*, and* $q$ *for a total of* $N=90000$ *simulations per model. Individual test p-values* $\leq.05$ *were considered significant. The G_BLUP_ model was not included in this comparison.*

|  | **Type I Error** | **Power** | | | |
| --- | --- | --- | --- | --- | --- |
|  | $\boldsymbol{\beta=0}$ | $\boldsymbol{\beta=0.125}$ | $\boldsymbol{\beta=0.25}$ | $\boldsymbol{\beta=0.375}$ | $\boldsymbol{\beta=0.5}$ |
| **MM_add_(X)** | 0.0502 | 0.3213 | 0.7744 | 0.9657 | 0.9980 |
| **MM_nestG_(X)** | 0.0520 | 0.3110 | 0.7600 | 0.9603 | 0.9970 |

**Figures**


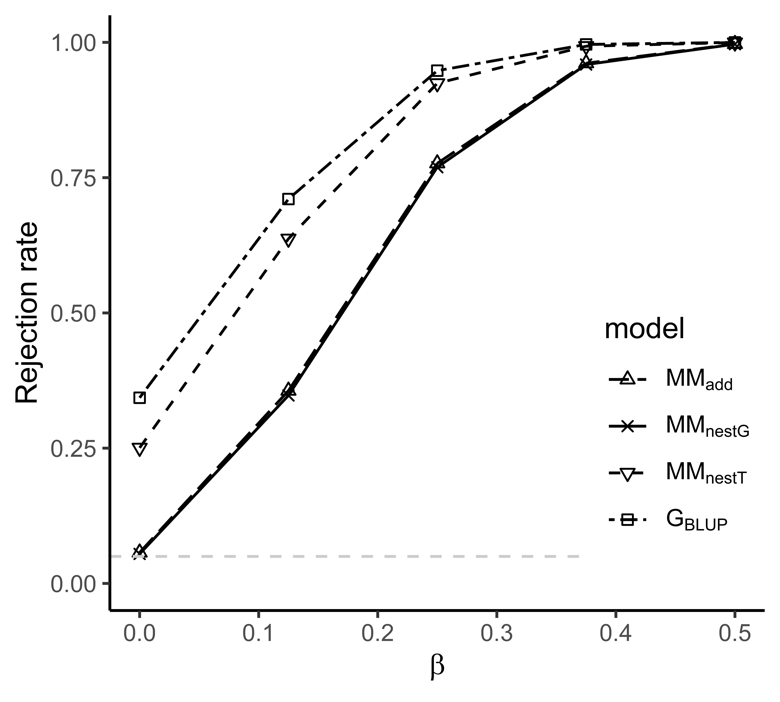


Figure E: Power curves of competing genomic association models without trait covariates, over true levels of $\beta$. The models MM_tfix_ and MM_full_ are not shown because they perform identically to MM_add_. Values are averaged over all levels of $\alpha$, $\phi$, and $q$ for a total of $N=90000$ simulations per model. Individual test p-values $\leq.05$ were considered significant.


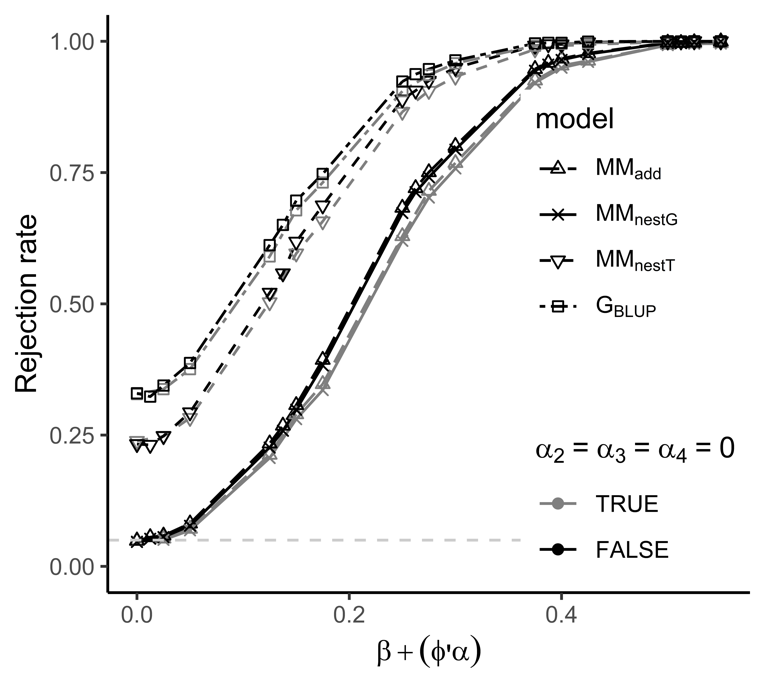


Figure F: Power curves of competing genomic association models without trait covariates, over levels of the total SNP effects $\beta+(\phi^{'}\alpha)$. The models MM_tfix_ and MM_full_ are not shown because they perform identically to MM_add_. Grey lines and points represent cases in which none of traits 2, 3, or 4 had any effect on the extended phenotype and black lines and points represent cases in which at least one of these traits had effects greater than 0. Values are also averaged over the levels of $q$ for a total of $N=6000$ simulations per model. Individual test p-values $\leq.05$ were considered significant.


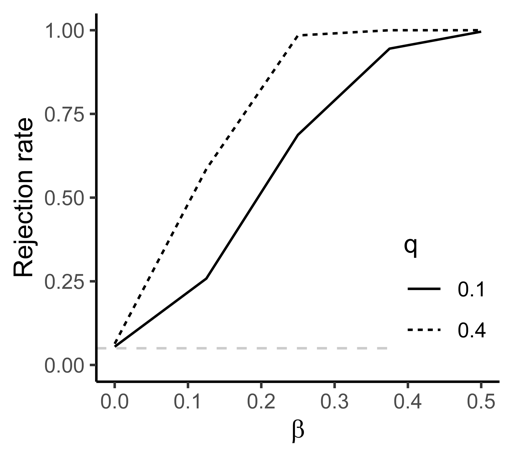


Figure G: Effect of the minor allele frequency $q$ on the power to detect genomic associations. The rejection rate of the MM_add_ model without trait covariates, is shown as a function of the SNP effect $\beta$, averaged over all levels of $\phi$ and $\alpha$, with a total $N=90000$ simulations. All other mixed models responded similarly to changes in $q$ but are not shown here for clarity.


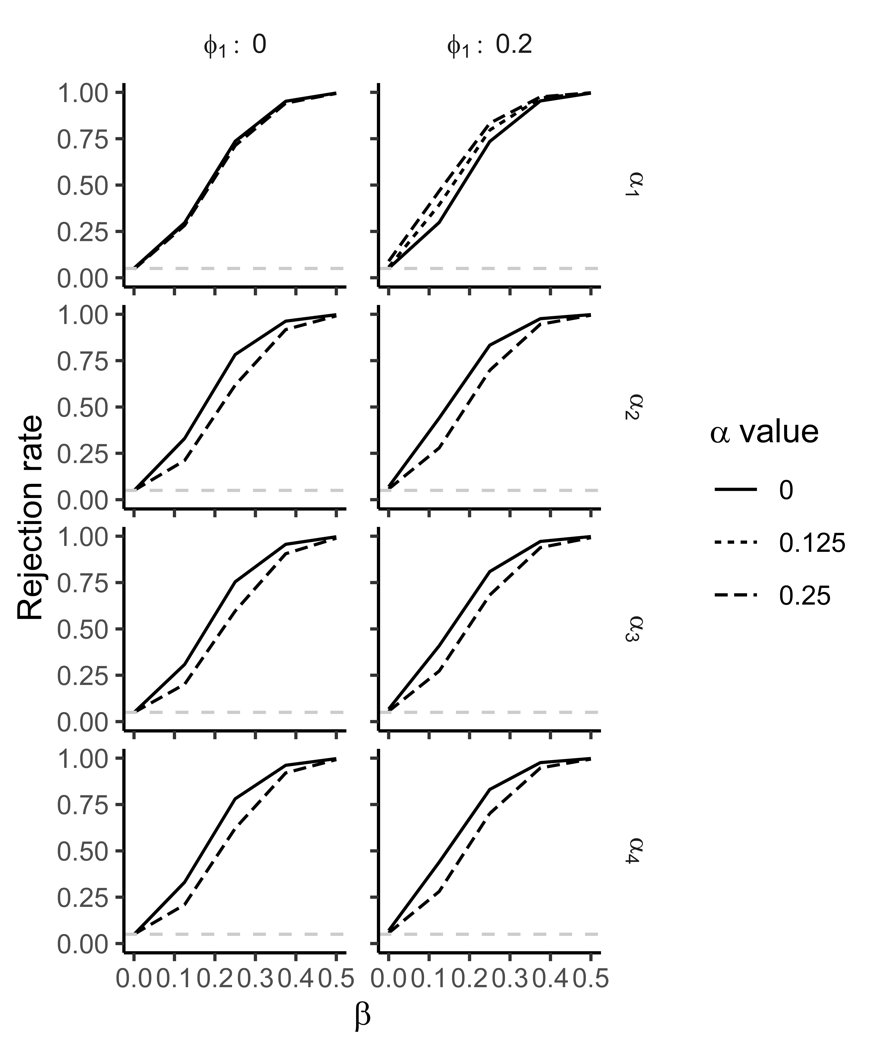


Figure H: Impact of indirect genetic effects and phenotypic effects on detecting genomic associations, for models without trait covariates. The rejection rate of the MM_add_ model is shown as a function of the SNP effect $\beta$ for different values of $\alpha_{i}$ for each trait *i*, and $\phi_{1}$, averaged across levels of minor allele frequency. Panels are divided into rows corresponding to the four different traits’ effects and columns corresponding to the indirect genetic effect on $Y$ through trait one. The different lines in a panel correspond to the tested levels of each trait effect $\alpha_{i}$. Results are based on $N=18000$ total simulations for each panel. All other mixed models responded similarly to changes in $\alpha$ and $\phi$ but are not shown here for clarity.

**
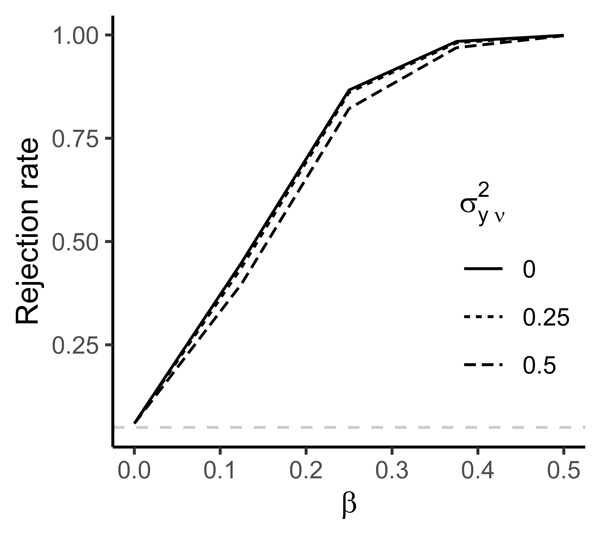
**

Figure I: Impact of the magnitude of the random interaction between genetic and temporal factors ($\sigma_{y\nu}^{2}$) for the MM_add_ genomic association model without trait covariates. The line different values of $\sigma_{y\nu}^{2}$ are represented by the different line types. Results are averaged over all levels of $\alpha$, $\phi$, and $q$. Patterns in other mixed-effects models are similar but are excluded for visual clarity.

**
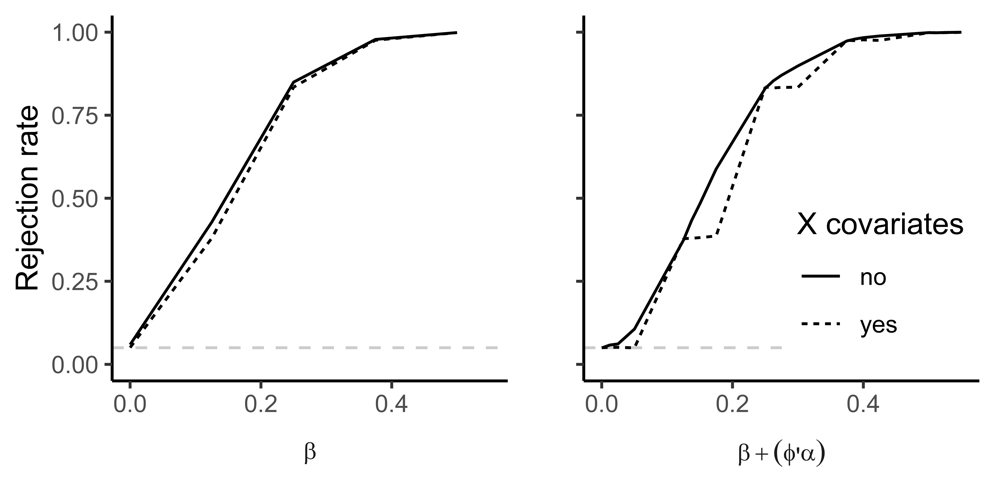
**

Figure J: Impact of including X covariates in the MM_add_ genomic association model. The left panel corresponds to the detection of parameter $\hat{\beta}$, given the true *indirect* effect $\beta$ (x-axis), for models with and without covariates. The right panel corresponds to detection of $\hat{\beta}$, given the true *total* SNP effect $\beta+\phi^{'}\alpha$. Solid lines represent the model fit without trait covariates and the dashed line represents the model including trait covariates. Results are averaged over all values of $q$. Patterns in other mixed-effects models are similar but are excluded for visual clarity.

**
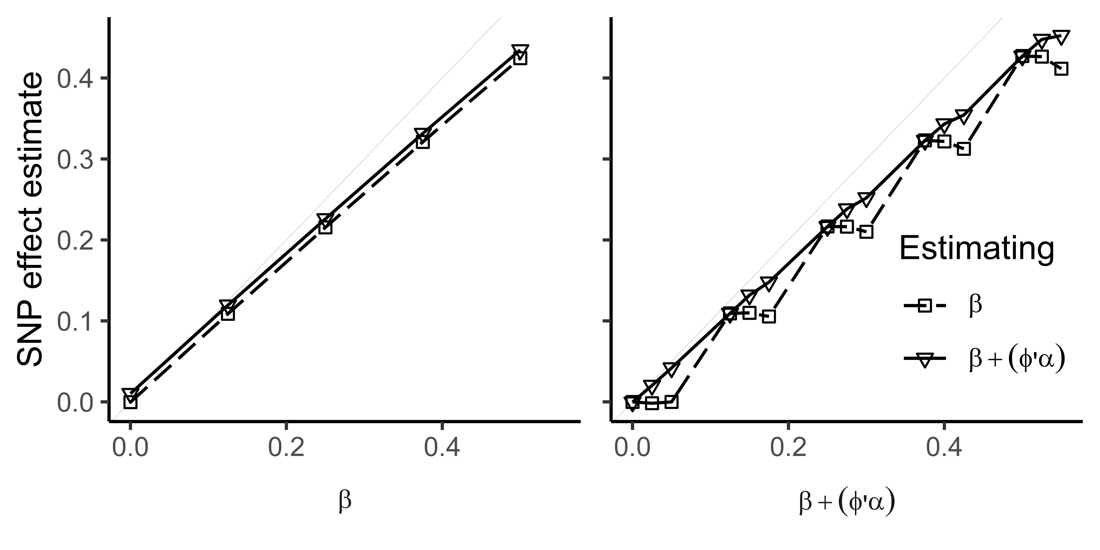
**

Figure K: Estimated SNP effects of MM_add_ with and without trait covariates. Triangles and solid lines represent the version of MM_add_ without trait covariates and squares and solid lines represent the model that includes trait covariates. The y-axis represents the average effect estimated from the model while the x-axis represents the actual effect. The left panel shows the estimated effect vs. the true *direct* SNP effect and the right panel shows estimated effects vs. the true *total* SNP effects. The model with covariates estimates the *direct* effect, while the model without covariates estimates the total effect. Patterns in other mixed-effects models are similar but are excluded for visual clarity.
