# Supplementary material for: Canopy insect communities are shaped by the genes and phenotypes of their aspen hosts
Source: PLoS One. 2025 Jul 17;20(7):e0327554. doi: 10.1371/journal.pone.0327554 (PMC12270107; doi:10.1371/journal.pone.0327554)
Supplement: S2 File — (DOCX) [file pone.0327554.s002.docx]

Supplemental Figures and Tables

# Supplemental Figures


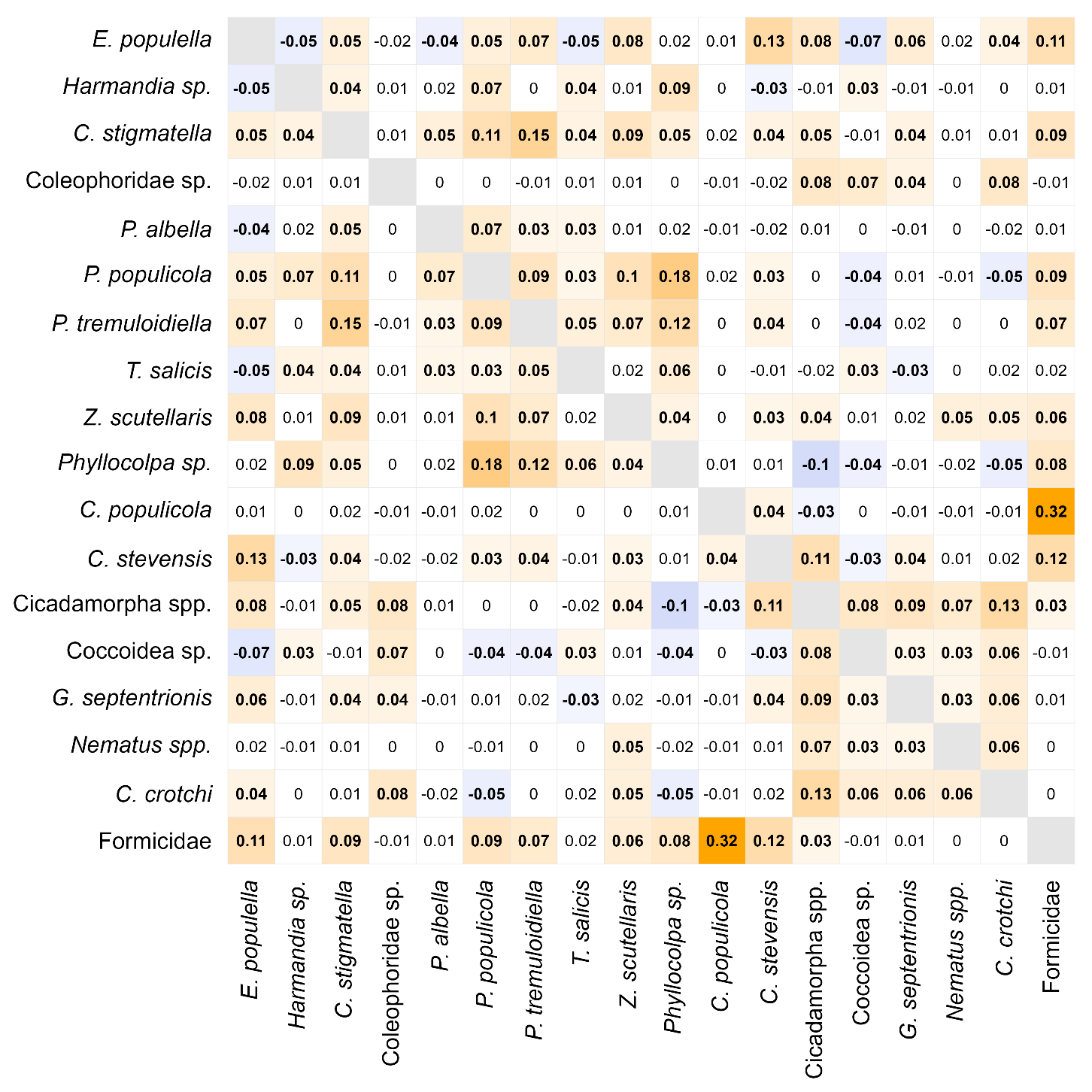


*Figure A*: Correlations among insect species. Warm colors correspond to positive correlations and cool colors to negative correlations. Color intensity corresponds to magnitude of the correlation. Cell values represent Pearson’s correlation coefficient, and statistically significant (p < 0.05) correlations are bolded.


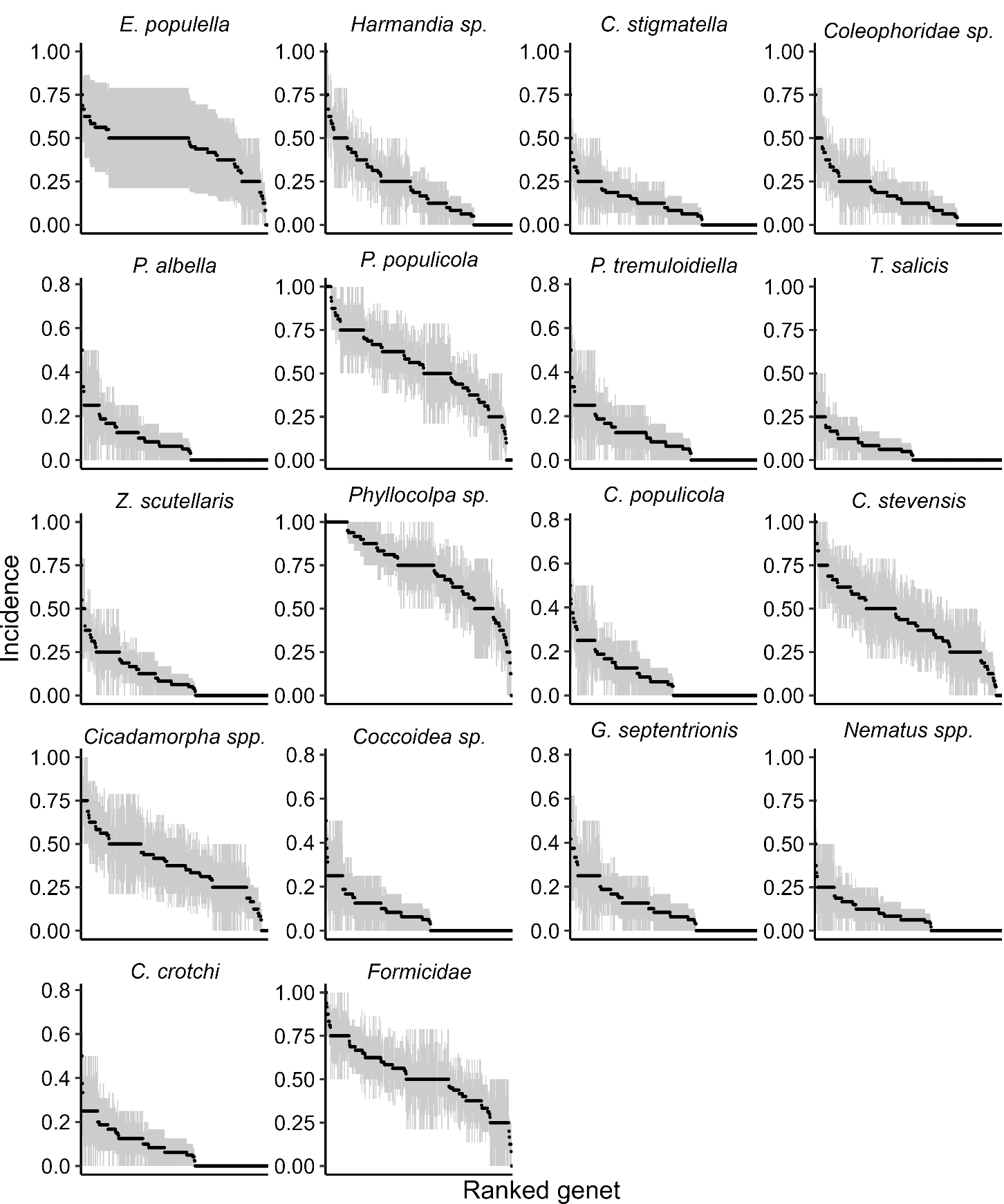


*Figure B*: Variation in common insect incidence among aspen genets. Points represent the proportion of trees within a genet on which an insect occurred during a survey event. Grey bars represent ± 1 standard deviation across survey events. Panels correspond to the different insect species. Probabilities were derived from the logistic mixed effects models.


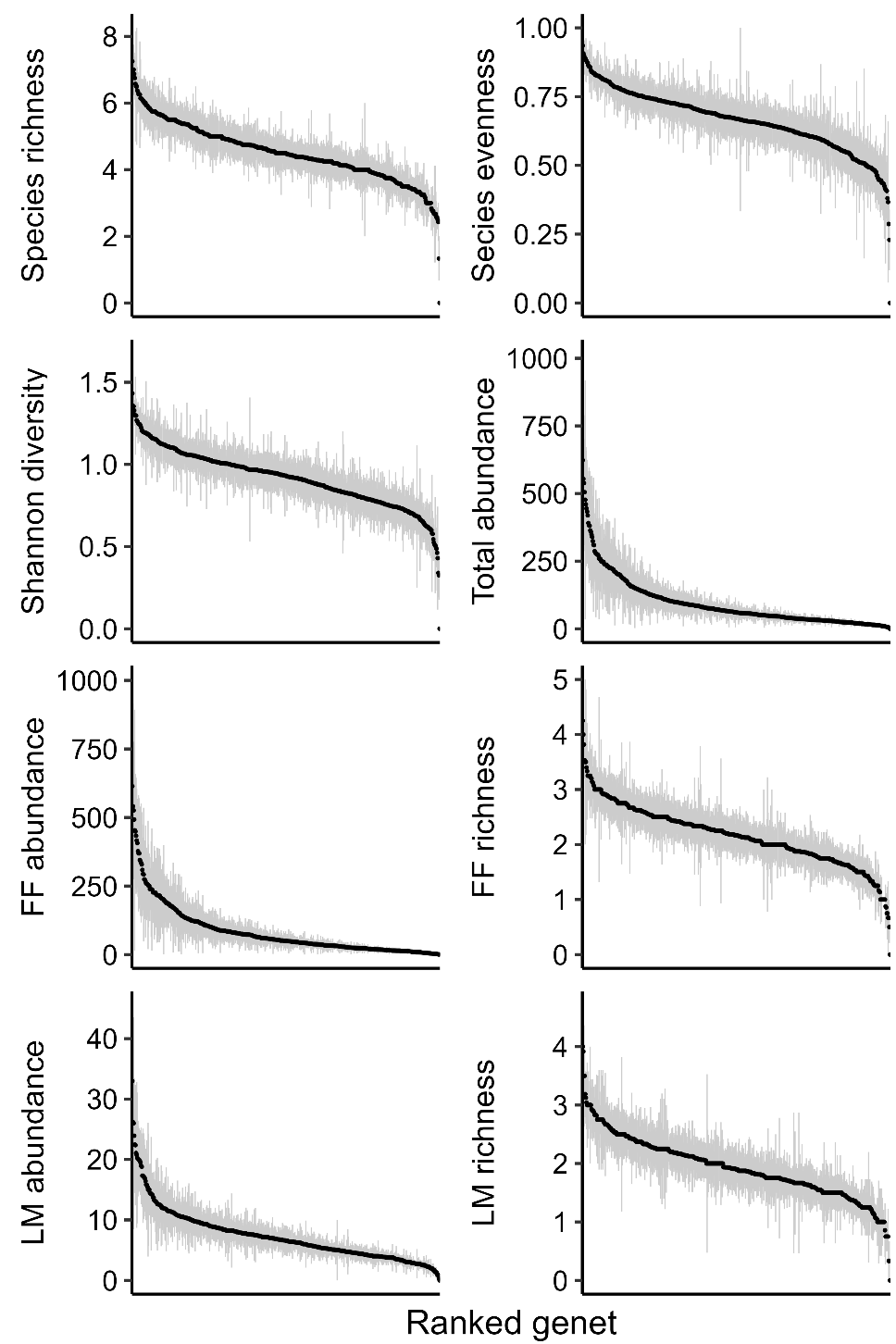


*Figure C*: Variation in community metrics among aspen genets. The top four panels correspond to whole-community metrics and the lower four correspond to free-feeding (FF) and leaf-modifying (LM) functional groups. Points represent genet trait averages and grey error bars represent ± 1 standard deviation. Genets are ordered by descending rank for each metric.


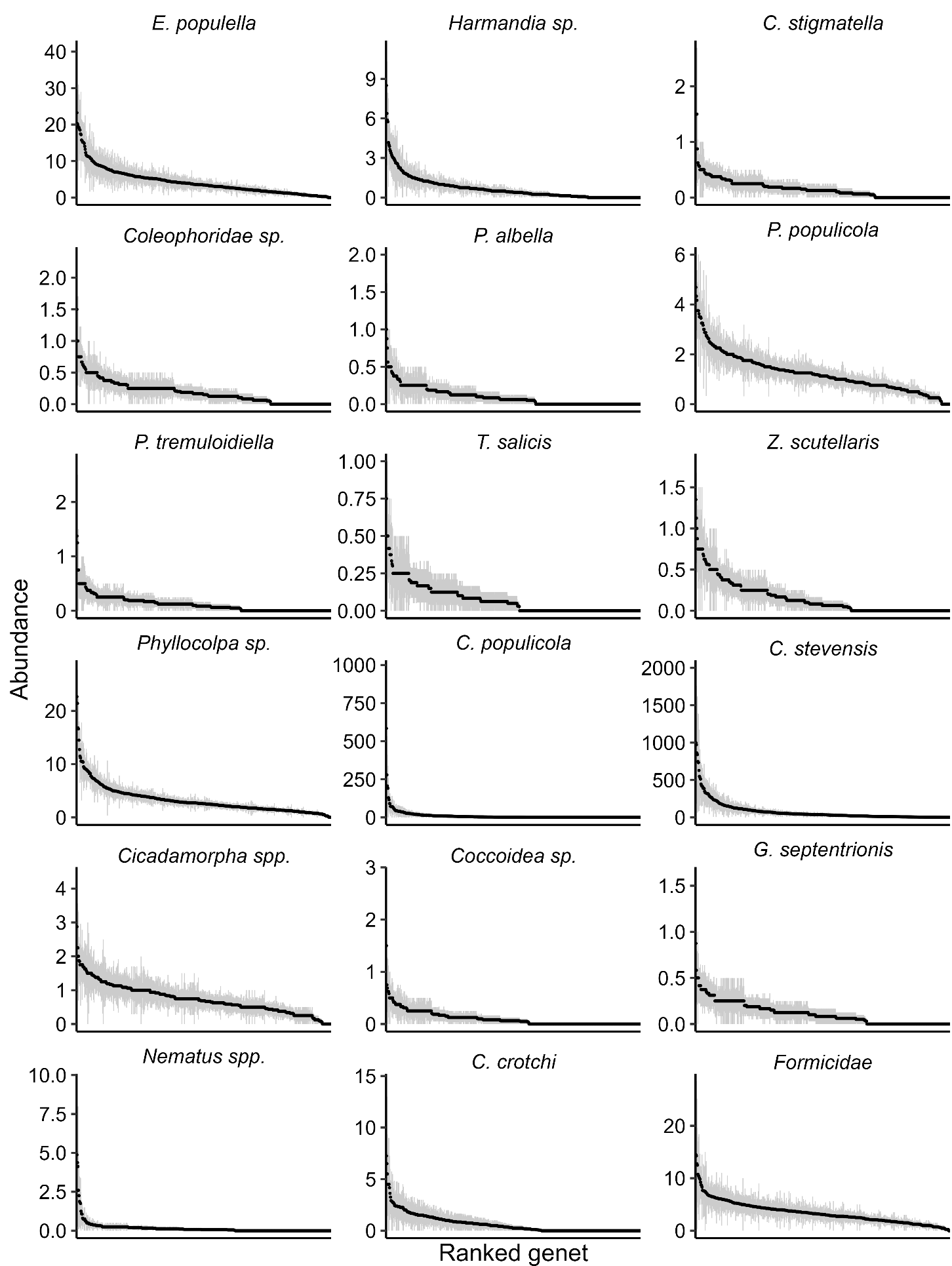


*Figure D*: Variation in common insect abundance among aspen genets. Points represent the average insect abundance on a genet and grey error bars represent ± 1 standard deviation. Genets are ordered by descending rank for each insect. Panels correspond to the different insect species.

**
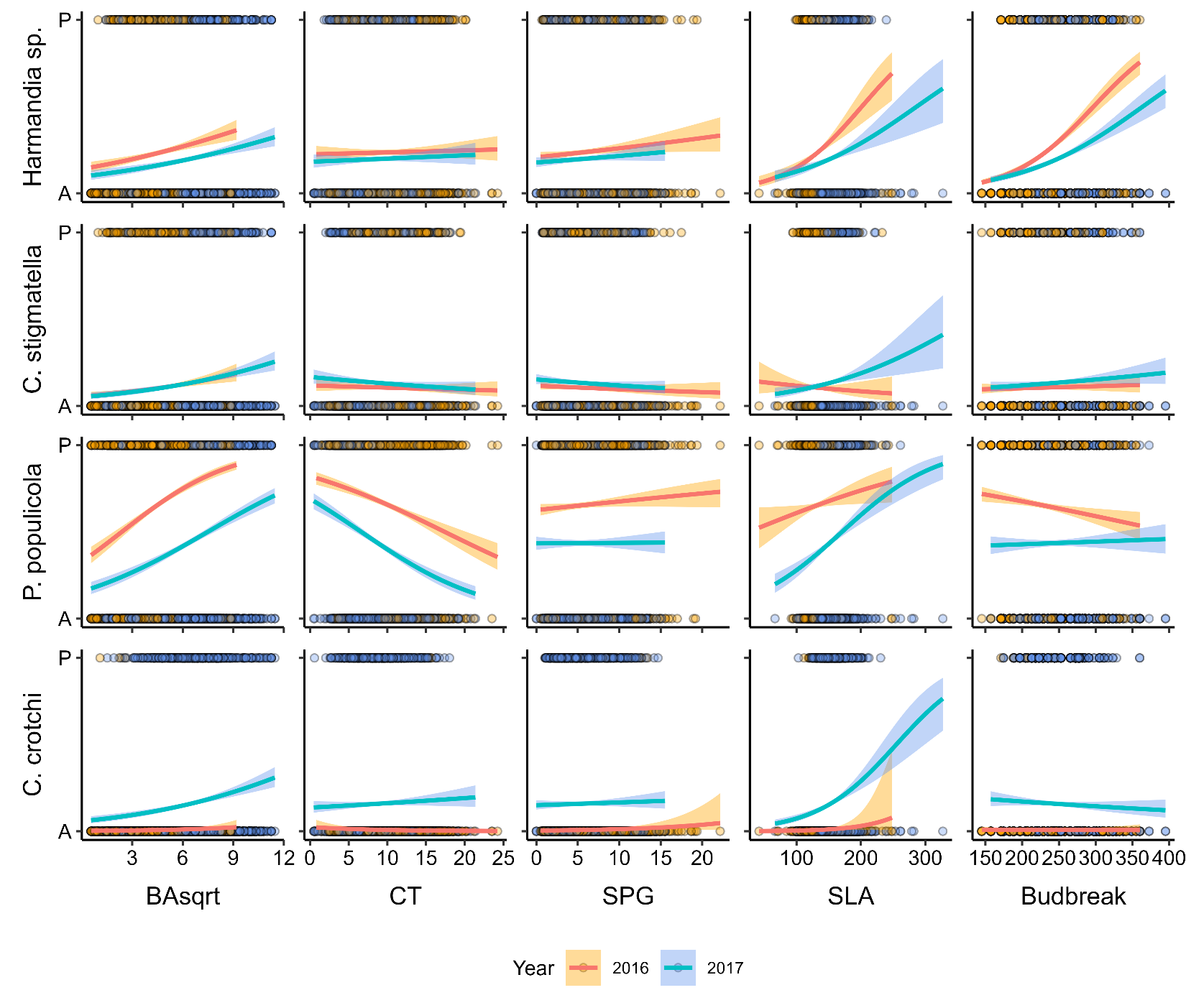
**

*Figure E*: Bivariate logistic regression of insect incidence (presence: P, absence: A) against key tree traits by year. A subset of five tree traits and four insects affected by those traits were selected as exemplary of the raw relationships between tree traits and incidence, before accounting for other traits and environmental factors. Abbreviations are BAsqrt: square root of basal area, CT: condensed tannins, SPG: salicinoid phenolic glycosides, and SLA: specific leaf area. BAsqrt is in cm, CT and SPG are in %, SLA is unitless, and budbreak is in degree days.

# Supplemental Tables

*Table A*: Genomic associations with aspen traits; Columns represent traits, associated SNP IDs, gene IDs, description of the known gene function, effect of the minor alleles on insect incidence, p-value, and Storey’s q-value. Missing gene and annotation entries indicate that the gene in which the SNP is located remains unknown.

| Trait | SNP | Gene | Annotation | Coefficient | p-value | q-value |
| --- | --- | --- | --- | --- | --- | --- |
| EFN | Potra000613:36082 | Potra000613g04664 | probable polygalacturonase | 0.2376 | 0.0000008 | 0.0897 |
| Flowers | Potra003360:9560 |  |  | 20.2735 | 0.0000883 | 0.1413 |
| Flowers | Potra002011:10034 |  |  | 22.9675 | 0.0000011 | 0.0176 |
| Flowers | Potra001830:1116 |  |  | 20.8740 | 0.0000247 | 0.0847 |
| Flowers | Potra179589:10982 | Potra179589g28026 | ABC transporter B family member 1;zinc finger protein 593 | 19.3534 | 0.0000349 | 0.0936 |
| Flowers | Potra003095:11323 |  |  | 17.2710 | 0.0000052 | 0.0478 |
| Flowers | Potra002370:11739 |  |  | 21.5176 | 0.0000194 | 0.0710 |
| Flowers | Potra002370:11789 |  |  | 19.8863 | 0.0000283 | 0.0864 |
| Flowers | Potra000831:14132 | Potra000831g06665 | protein ABHD11 isoform X1 | 22.0439 | 0.0000015 | 0.0176 |
| Flowers | Potra003722:14151 | Potra003722g22573 | Homoserine O-acetyltransferase | 14.6633 | 0.0000788 | 0.1331 |
| Flowers | Potra001465:15308 |  |  | 16.7313 | 0.0000616 | 0.1215 |
| Flowers | Potra001465:15381 |  |  | 17.5326 | 0.0000307 | 0.0864 |
| Flowers | Potra001465:15399 |  |  | 17.1997 | 0.0000404 | 0.0965 |
| Flowers | Potra002492:19040 | Potra002492g18832 | aluminum-activated malate transporter 12-like | 19.7561 | 0.0000438 | 0.1002 |
| Flowers | Potra000179:19529 | Potra000179g00698 | yae1 domain-containing protein 1-like isoform X1 | 20.6225 | 0.0000302 | 0.0864 |
| Flowers | Potra000655:23796 |  |  | 22.2036 | 0.0001101 | 0.1460 |
| Flowers | Potra002557:25018 | Potra002557g19269 | pentatricopeptide repeat-containing protein At1g08070, chloroplastic;tocopherol cyclase;tocopherol cyclase, chloroplastic | 19.0534 | 0.0000960 | 0.1460 |
| Flowers | Potra003994:25486 | Potra003994g24046 | UDP-glycosyltransferase 73C1-like | 19.8085 | 0.0000404 | 0.0965 |
| Flowers | Potra002243:28532 | Potra002243g17245 | phytoene dehydrogenase, chloroplastic/chromoplastic | 33.8471 | 0.0000000 | 0.0008 |
| Flowers | Potra001263:29158 | Potra001263g10903 | transcription factor bHLH137-like | 23.0577 | 0.0000329 | 0.0902 |
| Flowers | Potra001520:31078 | Potra001520g12656 | vam6/Vps39-like protein | 23.8243 | 0.0000014 | 0.0176 |
| Flowers | Potra002818:34295 | Potra002818g20041 | serine/arginine-rich splicing factor SR34A;IAA-amino acid hydrolase ILR1-like 5;IAA-amino acid hydrolase ILR1-like 3 | 19.2533 | 0.0001131 | 0.1460 |
| Flowers | Potra002818:34337 | Potra002818g20041 | serine/arginine-rich splicing factor SR34A;IAA-amino acid hydrolase ILR1-like 5;IAA-amino acid hydrolase ILR1-like 3 | 19.2533 | 0.0001131 | 0.1460 |
| Flowers | Potra000987:36334 | Potra000987g08153 | subtilisin-like protease SBT5.4 | 22.3181 | 0.0000177 | 0.0710 |
| Flowers | Potra003428:37219 | Potra003428g21635 | hypothetical protein POPTR_0009s08770g;serine/threonine-protein kinase TOR | 18.2227 | 0.0000622 | 0.1215 |
| Flowers | Potra000454:37262 | Potra000454g02582 | ABC transporter G family member 14-like;histone H1 | 26.0026 | 0.0000109 | 0.0682 |
| Flowers | Potra000759:38762 | Potra000759g05981 | protein SMG7L-like;B3 domain-containing transcription factor LEC2-like | 21.7284 | 0.0000137 | 0.0690 |
| Flowers | Potra003809:41142 | Potra003809g22941 | tubulin-folding cofactor A | 16.7806 | 0.0001171 | 0.1478 |
| Flowers | Potra002589:41363 | Potra002589g19466 | DEAD-box ATP-dependent RNA helicase 41 | 17.5329 | 0.0000068 | 0.0537 |
| Flowers | Potra002560:41388 | Potra002560g19293 | F-box/LRR-repeat protein At3g26922-like | 11.1633 | 0.0001030 | 0.1460 |
| Flowers | Potra002461:43136 | Potra002461g18651 | alkane hydroxylase MAH1-like | 21.1303 | 0.0001121 | 0.1460 |
| Flowers | Potra000522:43553 | Potra000522g03533 | putative G3BP-like protein | 19.7715 | 0.0000526 | 0.1112 |
| Flowers | Potra002005:2215 | Potra002005g15734 | IAA-amino acid hydrolase ILR1-like 4 | 28.2367 | 0.0000000 | 0.0008 |
| Flowers | Potra003941:49406 | Potra003941g23670 | bifunctional aspartokinase/homoserine dehydrogenase | 16.0874 | 0.0001037 | 0.1460 |
| Flowers | Potra003985:49461 | Potra003985g23998 | protein TRANSPARENT TESTA 16;protein TRANSPARENT TESTA 16-like | 22.2990 | 0.0000174 | 0.0710 |
| Flowers | Potra003985:49463 | Potra003985g23998 | protein TRANSPARENT TESTA 16;protein TRANSPARENT TESTA 16-like | 22.2990 | 0.0000174 | 0.0710 |
| Flowers | Potra003985:49501 | Potra003985g23998 | protein TRANSPARENT TESTA 16;protein TRANSPARENT TESTA 16-like | 22.2990 | 0.0000174 | 0.0710 |
| Flowers | Potra000571:50822 | Potra000571g04226 | nascent polypeptide-associated complex subunit alpha-like protein 2 | 10.6624 | 0.0000749 | 0.1331 |
| Flowers | Potra000354:51695 | Potra000354g01357 | putative uridine kinase C227.14 | 15.0237 | 0.0001052 | 0.1460 |
| Flowers | Potra003669:2337 | Potra003669g22380 | serine/threonine-protein kinase tricorner isoform X1 | 20.0184 | 0.0000453 | 0.1015 |
| Flowers | Potra002524:52825 | Potra002524g19049 | protein SABRE isoform X1;GATA transcription factor 24-like isoform X1 | 21.9435 | 0.0000527 | 0.1112 |
| Flowers | Potra003669:2441 | Potra003669g22380 | serine/threonine-protein kinase tricorner isoform X1 | 25.0984 | 0.0000018 | 0.0181 |
| Flowers | Potra001330:57400 | Potra001330g11428 | probable pectinesterase/pectinesterase inhibitor 21;geranylgeranyl transferase type-2 subunit beta 1-like isoform X1 | 25.3412 | 0.0000300 | 0.0864 |
| Flowers | Potra000489:59160 | Potra000489g03058 | CBL-interacting protein kinase 32;CBL-interacting protein kinase 32-like;CBL-interacting serine/threonine-protein kinase 3 isoform X2;probable peptide/nitrate transporter At3g43790 isoform X1 | 23.0257 | 0.0000786 | 0.1331 |
| Flowers | Potra000489:59180 | Potra000489g03058 | CBL-interacting protein kinase 32;CBL-interacting protein kinase 32-like;CBL-interacting serine/threonine-protein kinase 3 isoform X2;probable peptide/nitrate transporter At3g43790 isoform X1 | 23.0257 | 0.0000786 | 0.1331 |
| Flowers | Potra004010:61629 | Potra004010g24171 | BRASSINOSTEROID INSENSITIVE 1-associated receptor kinase 1-like isoform X2;BRASSINOSTEROID INSENSITIVE 1-associated receptor kinase 1;BRASSINOSTEROID INSENSITIVE 1-associated receptor kinase 1-like;nuclear transcription factor Y subunit B-1 | 22.2173 | 0.0000190 | 0.0710 |
| Flowers | Potra000574:65122 | Potra000574g04268 | protein trichome birefringence-like 31 | 20.1236 | 0.0000184 | 0.0710 |
| Flowers | Potra000574:65145 | Potra000574g04268 | protein trichome birefringence-like 31 | -10.2868 | 0.0000747 | 0.1331 |
| Flowers | Potra000487:67955 | Potra000487g03013 | 6,7-dimethyl-8-ribityllumazine synthase;serine/threonine-protein phosphatase PP2A-2 catalytic subunit | 26.7196 | 0.0000008 | 0.0176 |
| Flowers | Potra000487:69884 | Potra000487g03013 | 6,7-dimethyl-8-ribityllumazine synthase;serine/threonine-protein phosphatase PP2A-2 catalytic subunit;phytoene synthase 2, chloroplastic-like | 22.7744 | 0.0000111 | 0.0682 |
| Flowers | Potra001237:69981 | Potra001237g10614 | luc7-like protein 3;aldo-keto reductase family 4 member C9 | 23.2779 | 0.0000368 | 0.0939 |
| Flowers | Potra001184:74346 | Potra001184g10259 | bidirectional sugar transporter SWEET15-like;leucine-rich repeat protein 1-like | 23.4909 | 0.0000653 | 0.1236 |
| Flowers | Potra002914:2765 |  |  | 14.5362 | 0.0000888 | 0.1413 |
| Flowers | Potra003598:2767 |  |  | 27.1403 | 0.0000117 | 0.0682 |
| Flowers | Potra000791:86315 | Potra000791g06264 | actin-related protein 8 | 26.4287 | 0.0000274 | 0.0864 |
| Flowers | Potra001712:86971 | Potra001712g13935 | protein CASP | 20.4560 | 0.0001016 | 0.1460 |
| Flowers | Potra001042:91473 | Potra001042g08802 | 2,4-dichlorophenol 6-monooxygenase isoform X1;2,4-dichlorophenol 6-monooxygenase;flocculation protein FLO11 | 20.7520 | 0.0001094 | 0.1460 |
| Flowers | Potra000991:91852 | Potra000991g08205 | transcription factor MYB3R-3 isoform X1 | 20.7590 | 0.0000274 | 0.0864 |
| Flowers | Potra000973:92429 | Potra000973g08033 | DNA-directed RNA polymerase III subunit RPC3;lipase class 3 family protein | 17.9285 | 0.0001034 | 0.1460 |
| Flowers | Potra001256:96915 |  |  | 25.0380 | 0.0000360 | 0.0939 |
| Flowers | Potra001632:100665 |  |  | 22.3602 | 0.0001154 | 0.1474 |
| Flowers | Potra000458:105260 | Potra000458g02641 | triacylglycerol lipase 2-like;triacylglycerol lipase 2;cytochrome P450 94B3 | 32.7805 | 0.0000000 | 0.0001 |
| Flowers | Potra001115:107841 | Potra001115g09792 | —NA—;probable beta-1,3-galactosyltransferase 2 isoform X1;probable beta-1,3-galactosyltransferase 2 | 18.8279 | 0.0000753 | 0.1331 |
| Flowers | Potra004004:111201 | Potra004004g24122 | glycoside hydrolase 9C1 | 17.4155 | 0.0000086 | 0.0629 |
| Flowers | Potra002010:112137 | Potra002010g15755 | serine/threonine-protein kinase rio1-like | 18.9635 | 0.0000550 | 0.1139 |
| Flowers | Potra002010:112138 | Potra002010g15755 | serine/threonine-protein kinase rio1-like | 17.7557 | 0.0000414 | 0.0967 |
| Flowers | Potra001016:131314 | Potra001016g08480 | chaperone protein dnaJ 10 | 22.5543 | 0.0000144 | 0.0690 |
| Flowers | Potra001016:131315 | Potra001016g08480 | chaperone protein dnaJ 10 | 22.5543 | 0.0000144 | 0.0690 |
| Flowers | Potra001016:131319 | Potra001016g08480 | chaperone protein dnaJ 10 | 24.2872 | 0.0000302 | 0.0864 |
| Flowers | Potra001655:132991 | Potra001655g13592 | beta-glucosidase 12-like | 20.1965 | 0.0000016 | 0.0176 |
| Flowers | Potra000406:159495 | Potra000406g01981 | CASP-like protein 1D1 | 28.7563 | 0.0000015 | 0.0176 |
| Flowers | Potra000417:162754 | Potra000417g02120 | PREDICTED: uncharacterized protein LOC105116303 isoform X1;PREDICTED: uncharacterized protein LOC105116303 isoform X4;PREDICTED: uncharacterized protein LOC105116303 isoform X3 | 21.2563 | 0.0000786 | 0.1331 |
| Flowers | Potra002021:3235 | Potra002021g15846 | cytochrome P450 724B1-like;subtilisin-like protease SBT5.3 | 20.9452 | 0.0000479 | 0.1052 |
| Flowers | Potra000346:182411 | Potra000346g01235 | dnaJ homolog subfamily B member 6 isoform X2 | 22.6748 | 0.0000631 | 0.1215 |
| Flowers | Potra000417:191626 | Potra000417g02122 | ankyrin repeat domain-containing protein EMB506, chloroplastic;NA;ATP-dependent Clp protease proteolytic subunit-related protein 1, chloroplastic | 21.4143 | 0.0000585 | 0.1189 |
| Flowers | Potra002046:3840 |  |  | 25.1108 | 0.0000068 | 0.0537 |
| Flowers | Potra188252:4042 | Potra188252g28584 | auxin-responsive family protein;NA | 23.6016 | 0.0000118 | 0.0682 |
| Flowers | Potra195163:731 |  |  | 24.9127 | 0.0000853 | 0.1413 |
| Flowers | Potra002021:752 | Potra002021g15847 | subtilisin-like protease SBT5.3 | 20.5117 | 0.0000211 | 0.0748 |
| Flowers | Potra006505:5167 | Potra006505g25700 | probable galacturonosyltransferase 11;probable galacturonosyltransferase 11 isoform X1 | 14.2891 | 0.0000991 | 0.1460 |
| Flowers | Potra000327:5996 | Potra000327g01147 | BTB/POZ and MATH domain-containing protein 2-like | 22.3425 | 0.0000879 | 0.1413 |
| Flowers | Potra002304:6073 | Potra002304g17573 | haloacid dehalogenase-like hydrolase domain-containing protein 3 | 15.6213 | 0.0000906 | 0.1421 |
| Flowers | Potra001421:6293 | Potra001421g12026 | embryo-specific family protein;embryo-specific protein ATS3B-like | 18.9137 | 0.0000986 | 0.1460 |
| Flowers | Potra009102:6556 | Potra009102g26288 | putative H/ACA ribonucleoprotein complex subunit 1-like protein 1 | 14.7825 | 0.0000933 | 0.1443 |
| Flowers | Potra006505:7038 | Potra006505g25700 | probable galacturonosyltransferase 11;probable galacturonosyltransferase 11 isoform X1 | 14.3837 | 0.0001098 | 0.1460 |
| Flowers | Potra182098:7279 | Potra182098g28206 | flavin-containing monooxygenase FMO GS-OX-like 9 | 13.4844 | 0.0000129 | 0.0690 |
| Flowers | Potra000817:8282 | Potra000817g06506 | hypothetical protein POPTR_0001s00840g | 20.6756 | 0.0000376 | 0.0939 |
| Flowers | Potra000817:8915 | Potra000817g06506 | hypothetical protein POPTR_0001s00840g | 24.4507 | 0.0000002 | 0.0064 |
| Budbreak | Potra182540:1252 |  |  | -11.2187 | 0.0000066 | 0.0646 |
| Budbreak | Potra001174:13069 | Potra001174g10169 | dr1-associated corepressor homolog;11-oxo-beta-amyrin 30-oxidase-like;cytochrome P450 4X1 isoform X1 | 20.2228 | 0.0000165 | 0.1016 |
| Budbreak | Potra002201:22864 | Potra002201g16952 | histidine kinase 3 | 12.1206 | 0.0000049 | 0.0646 |
| Budbreak | Potra002720:23256 | Potra002720g19828 | kinesin heavy chain isoform X2;kinesin-like protein KIN-6 isoform X1;hypothetical protein POPTR_0013s06510g | 18.7558 | 0.0000235 | 0.1298 |
| Budbreak | Potra003186:38359 | Potra003186g20928 | NDR1/HIN1-like protein 1;transmembrane protein 53;Transmembrane protein 53;transmembrane protein 53-like | 12.4109 | 0.0000007 | 0.0469 |
| Budbreak | Potra003186:38398 | Potra003186g20928 | NDR1/HIN1-like protein 1;transmembrane protein 53;Transmembrane protein 53;transmembrane protein 53-like | 12.0553 | 0.0000017 | 0.0476 |
| Budbreak | Potra003186:38401 | Potra003186g20928 | NDR1/HIN1-like protein 1;transmembrane protein 53;Transmembrane protein 53;transmembrane protein 53-like | 12.1977 | 0.0000008 | 0.0469 |
| Budbreak | Potra003186:38410 | Potra003186g20928 | NDR1/HIN1-like protein 1;transmembrane protein 53;Transmembrane protein 53;transmembrane protein 53-like | 11.9429 | 0.0000015 | 0.0476 |
| Budbreak | Potra003809:46937 | Potra003809g22943 | —NA— | -10.4263 | 0.0000076 | 0.0646 |
| Budbreak | Potra002580:63210 | Potra002580g19401 | transcription repressor OFP17 | 15.0747 | 0.0000072 | 0.0646 |
| Budbreak | Potra167680:2612 | Potra167680g27295 | uncharacterized GPI-anchored protein At4g28100 | 14.1312 | 0.0000034 | 0.0633 |
| Budbreak | Potra167680:2613 | Potra167680g27295 | uncharacterized GPI-anchored protein At4g28100 | 14.1312 | 0.0000034 | 0.0633 |
| Budbreak | Potra002153:69635 | Potra002153g16652 | mediator of RNA polymerase II transcription subunit 7a;mediator of RNA polymerase II transcription subunit 7a-like;lipid phosphate phosphatase delta-like | 11.1288 | 0.0000107 | 0.0809 |
| Budbreak | Potra000434:171992 | Potra000434g02333 | probable ubiquitin-like-specific protease 2B isoform X1;Phosphoglucose isomerase (PGI) | 11.4500 | 0.0000274 | 0.1380 |
| Budbreak | Potra004013:5135 | Potra004013g24216 | dicarboxylate transporter 2.1, chloroplastic-like;DUF946 domain-containing protein | -9.6255 | 0.0000117 | 0.0809 |
| Budbreak | Potra001819:5495 | Potra001819g14674 | endonuclease III homolog 1, chloroplastic isoform X1 | 18.7605 | 0.0000047 | 0.0646 |
| Budbreak | Potra189842:6934 | Potra189842g28721 | protein MEI2-like 2;NA | 14.0280 | 0.0000257 | 0.1355 |
| Budbreak | Potra000831:6977 |  |  | -9.4821 | 0.0000062 | 0.0646 |
| Budbreak | Potra000831:6986 |  |  | -9.4821 | 0.0000062 | 0.0646 |
| Budbreak | Potra000831:7047 |  |  | -9.2276 | 0.0000154 | 0.1003 |
| Budbreak | Potra000831:7054 |  |  | -9.3455 | 0.0000114 | 0.0809 |
| Budbreak | Potra000831:8489 |  |  | -9.3367 | 0.0000184 | 0.1072 |
